# The effect of pharmacist-led interventions on the appropriateness and clinical outcomes of anticoagulant therapy: a systematic review and meta-analysis

**DOI:** 10.1093/ehjqcco/qcae045

**Published:** 2024-07-13

**Authors:** Belayneh Kefale, Gregory M Peterson, Corinne Mirkazemi, Woldesellassie M Bezabhe

**Affiliations:** School of Pharmacy and Pharmacology, College of Health and Medicine, University of Tasmania, Private Bag 26, Hobart, Tasmania 7000, Australia; Clinical Pharmacy Unit and Research Team, Department of Pharmacy, College of Medicine and Health Sciences, Bahir Dar University, Bahir Dar, Ethiopia; School of Pharmacy and Pharmacology, College of Health and Medicine, University of Tasmania, Private Bag 26, Hobart, Tasmania 7000, Australia; School of Pharmacy and Pharmacology, College of Health and Medicine, University of Tasmania, Private Bag 26, Hobart, Tasmania 7000, Australia; School of Pharmacy and Pharmacology, College of Health and Medicine, University of Tasmania, Private Bag 26, Hobart, Tasmania 7000, Australia

**Keywords:** Anticoagulant therapy, Pharmacist-led interventions, Atrial fibrillation, Bleeding, Venous thromboembolism

## Abstract

**Aim:**

Although pharmacist-led interventions in anticoagulant (AC) therapy are widely accepted, there is a lack of evidence comparing their effectiveness with usual care in terms of AC therapy appropriateness and clinical outcomes. We aimed to estimate the comparative effectiveness of pharmacist-led interventions on the appropriateness and clinical outcomes of AC therapy.

**Methods and results:**

Adhering to the PRISMA guidelines, we searched PubMed, EMBASE, and Scopus databases to identify randomized controlled trials and quasi-experimental and cohort studies published between 2010 and 2023. A random-effects model was used to calculate pooled intervention effects. We assessed heterogeneity (using Higgins’ *I*^2^ and Cochran's *Q*) and publication bias (using Egger's test, the trim-and-fill method, and visualization of the funnel plot). In total, 35 studies involving 10 374 patients in the intervention groups and 11 840 in the control groups were included. The pharmacist-led interventions significantly improved the appropriateness of AC therapy [odds ratio (OR): 3.43, 95% confidence interval (CI): 2.33–5.06, *P* < 0.01]. They significantly decreased total bleeding [relative risk (RR): 0.75, 95% CI: 0.58–0.96, *P* = 0.03) and hospitalization or readmission (RR: 0.64, 95% CI: 0.41–0.99, *P* = 0.04). However, the impact of the pharmacist-led interventions on thromboembolic events (RR: 0.69, 95% CI: 0.46–1.02, *P* = 0.07) and mortality (RR: 0.76, 95% CI: 0.51–1.13, *P* = 0.17) was not significant.

**Conclusion:**

Pharmacist-led interventions demonstrated superior outcomes in optimizing AC therapy compared with usual care. Further research is needed to evaluate pharmacist-led interventions’ cost-effectiveness and long-term sustainability.

**PROSPERO registration number:** CRD42023487362.

## Graphical Abstract

**Graphical Abstract:**
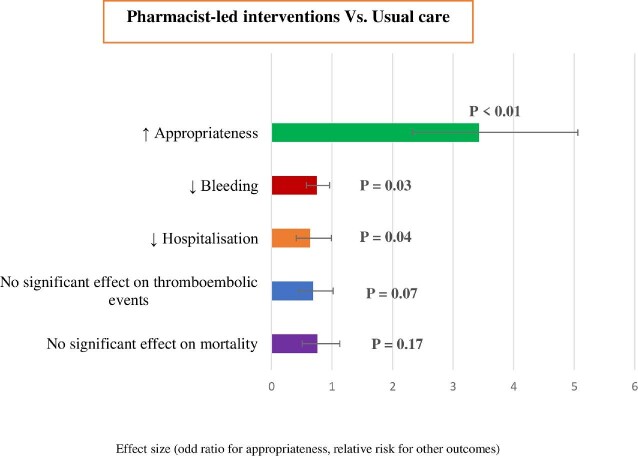

Key learning points **What is already known**

- Anticoagulants are high-risk medications associated with serious adverse drug events, even when adhering to clinical guidelines.

**What this study adds**

- Our systematic review and meta-analysis, the first of its kind, evaluate pharmacy-led interventions’ effectiveness on anticoagulant therapy appropriateness. Our findings show that patients under pharmacist-led interventions were over three times more likely to receive appropriate anticoagulant therapy.
- In our meta-analysis, we found pharmacist-led interventions were also significantly associated with reduced total bleeding and hospitalizations or readmission.
- This study provides evidence for the potential implementation of pharmacist-led interventions for improving the appropriateness of anticoagulant therapy and mitigating the risk of bleeding and hospitalizations.

## Introduction

Anticoagulant (AC) therapy is commonly used in patients with chronic atrial fibrillation (AF) or at risk of or experiencing venous thromboembolism (VTE).^1–4^ Despite the clear benefits of AC therapy in reducing and treating thrombotic events, ACs are classified as high-risk medications.^5,6^ This is because they are associated with serious adverse drug events (ADEs), even when used in accordance with clinical practice guidelines.^7–9^ Bleeding is the main ADE with AC treatment^10^ and is also a cause of AC discontinuation in up to 50% of patients.^11^ Patients taking oral anticoagulants (OACs) have up to an eight-fold increase in the risk of intracranial bleeding.^12,13^

Previous reviews have found that 8–29% of patients with indications for AC therapy receive inappropriate direct-acting oral anticoagulant (DOAC) prescriptions.^14,15^ Moreover, the prescribing of OACs for stroke prevention in patients with AF often does not comply with guidelines, resulting in underuse in high-risk patients and overuse in low-risk ones.^16–19^ The complexity of AC therapy has led to the development of pharmacist-led services to enhance patient outcomes through monitoring, dosage adjustment, and early identification of bleeding and VTE risk factors.^20,21^ Pharmacist-led interventions may improve medication use outcomes and patients’ quality of life^22–24^ and prevent medication-related harm.^22,25,26^

However, to our knowledge, no recent comprehensive reviews have evaluated the impact of pharmacist-led interventions on AC therapy appropriateness. Moreover, previous reviews on bleeding and thromboembolic events have been inconsistent in their findings and based on studies that predominantly included patients treated with warfarin.^27,28^ For example, Zhou *et al*.’s^28^ review revealed no significant difference in bleeding and thromboembolic events between pharmacist-managed care and usual care; however, reviews by Hou *et al.*^27^ and Lee *et al*.^25^ found that pharmacist-led AC therapy compared with usual care reduced bleeding risk and mortality, respectively. Notably, these reviews had important limitations, such as the small number of participants in most included studies.^25^ Furthermore, their findings predominantly reflected the pre-DOAC era, with some post-DOAC studies that also focused solely on patients managed with warfarin,^27,28^ despite warfarin being largely replaced by DOACs for stroke and VTE prevention.^29^ Therefore, this systematic review aimed to evaluate the effect of pharmacist-led interventions on the appropriateness and clinical outcomes of AC therapy, focusing especially on the period following the introduction of DOACs into practice.

## Methods

### Protocol

The protocol is available at PROSPERO (CRD42023487362).^30^ The findings are reported following the guidelines outlined in the Preferred Reporting Items for Systematic Reviews and Meta-Analyses (PRISMA) 2020 statement.^31^

### Data sources and search strategy

We searched PubMed, EMBASE, and Scopus for articles utilizing data since 2010 and published between 1 January 2010 and 24 November 2023 (updated on 23 December 2023). This time frame (2010 onwards) was chosen to focus on the post-DOAC era. The search strategy (developed in consultation with a research librarian) combined MeSH terms and keywords related to ACs, pharmacist-led intervention, AF, and VTE, as outlined in Supplement 1. We performed citation analysis by searching Google Scholar and the reference lists of the included studies. Study authors were contacted for additional data where necessary.

### Study selection criteria

All identified records were imported into Covidence software, where duplicates were removed. Two authors (B.K. and W.B.) independently reviewed each title and abstract; discrepancies were resolved with a third author (G.P.) by discussion and adjudication. All studies marked for possible inclusion underwent independent full-text review by two reviewers (B.K. reviewed all articles and W.B. and G.P. half each). Exclusion reasons were documented.

### Inclusion criteria

We included randomized controlled trials (RCTs), quasi-experimental (pre/post-intervention), and cohort studies that examined the effectiveness of pharmacist-led interventions compared with usual or physician-led care (both hereafter referred to as UC) in adults (aged ≥18 years) with AF or at risk of or having experienced VTE. Pharmacist-led interventions were defined as those in which pharmacists led an intervention to enhance AC therapy appropriateness and safety.

The primary outcomes of interest were as follows: (i) AC therapy appropriateness, evaluated based on evidence-based guidelines [e.g. American College of Clinical Pharmacy,^32,33^ summaries of product characteristics,^34–36^ country-specific guidelines,^37,38^ pharmacy-driven local protocol/guidelines^39–44^], and (ii) clinical outcomes, including thromboembolic events [systemic embolism, stroke, transient ischaemic attack, and myocardial infarction], bleeding events (major bleeding, minor or clinically significant non-major bleeding), and mortality. Hospitalization or readmission to hospital was a secondary outcome of interest.

### Exclusion criteria

We excluded studies focused on pharmacists’ perceptions, attitudes, views, knowledge, and awareness towards AC therapy; studies not available in English; studies with interventions not predominantly driven by a pharmacist; and theses, editorials, commentaries, conference abstracts, opinions, study protocols, guidelines, and qualitative studies.

### Quality assessment

We used the JBI Critical Appraisal Checklists to assess the quality of RCTs, quasi-experimental studies,^45^ and cohort studies.^46^ Two authors performed the appraisal (B.K. appraised all studies, and W.B., G.P., and C.M. appraised one-third each), and a third author resolved conflicts. The percentage of positively (yes) answered questions was calculated for each included study. The quality of evidence was ranked as high (score >70%), moderate (score 50–70%), or low (score <50%).^47^

### Data extraction

One author (B.K.) extracted data (using a standardized data extraction form), which was independently checked by a second author (W.B.); discrepancies were resolved via consensus. We extracted the author's name, country, publication year, study design and setting, participant characteristics, a description of the pharmacists’ intervention, outcome measures (e.g. number of patients with appropriate AC therapy), the type of ACs used and their indications, and a summary of key findings.

### Data synthesis and statistical analysis

Characteristics of patients and studies were summarized using descriptive statistics. We performed a meta-analysis for all outcomes using a random-effects model^48^ with an odds ratio (OR) or relative risk (RR) with 95% confidence intervals (CIs). Effect estimates were reported as ORs to compare the pooled appropriateness of AC therapy and as RRs to compare pooled events of clinical outcomes (bleeding, thromboembolic, mortality, and hospitalization or readmission) between the pharmacy-led and control groups. Han *et al.*^44^ reported two separate outcomes: the appropriateness of (i) AC discontinuation before endoscopy and (ii) AC bridging therapy. In our meta-analysis, we handled these as two independent studies. Any data not suitable for meta-analysis were narratively described.

Heterogeneity among studies was assessed using Higgins’ *I*^2^ and Cochran's *Q*.^49^ Using the *I*^2^ scale, heterogeneity is rated as low (0–25%), moderate (25–50%), substantial (50–75%), or considerable (>75%).^50^ Publication bias was assessed for all outcomes except mortality (as the number of studies was < 10) using Egger's test, the trim-and-fill method, and visualization of the funnel plot.^51,52^ We assessed the potential sources of heterogeneity using subgroup analyses (based on study design, region, and severity of bleeding) and leave-one-out analyses. The leave-one-out sensitivity analyses were performed by sequentially excluding each study to check its effect on the overall pooled OR or RR estimates for each outcome (AC appropriateness, bleeding events, thromboembolic events, and hospitalization or readmission). The statistical significance cut-off was *P* < 0.05. Analysis was conducted with STATA (Version 18.0; StataCorp, College Station, TX, USA).

## Results

### Study selection

**Figure 1:**
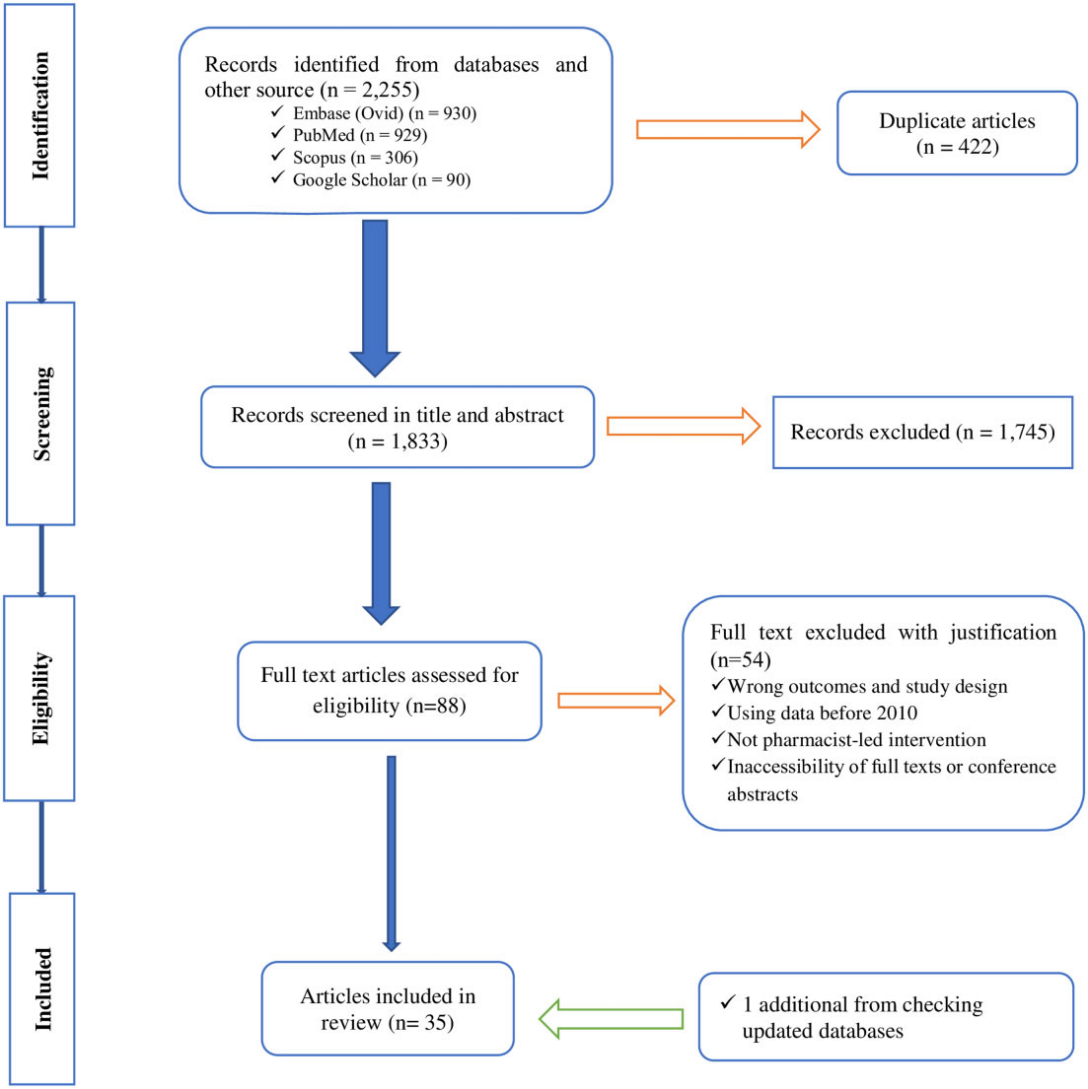
PRISMA flowchart of the study selection process and reasons for study exclusion.

We identified 2255 articles from the databases, of which 1833 unique studies were screened. Among these, 35 studies were included in the qualitative synthesis and meta-analysis. *Figure 1* summarizes the search results and reasons for exclusion.

### Studies and participants’ characteristics

The 35 studies included 10 374 individual patients in the intervention group (range: 17–4939) and 11 840 in the control group (range: 17–6182). The study settings were mainly hospitals [29 (83%) studies],^32,33,36–43,53–71^ and the participants’ mean ages ranged from 42 to 86 years. Study designs were varied: 5 were RCTs,^55,57,61,63,72^ 16 were quasi-experimental (pre/post-intervention),^32–34,36–40,42,53,54,56,58–60,62^ and 14 were cohort studies.^35,41,43,44,64–71,73,74^ Fourteen were conducted in Asia (5 in China,^43,56,57,63,64^ 3 in Japan,^65–67^ 2 in India,^32,61^ and one each in Taiwan,^54^ Malaysia,^40^ Saudi Arabia,^69^ and Lebanon^55^); 11 in North America (all in the USA)^35,39,41,44,59,62,68,70,71,73,74^; 6 in Europe (Belgium,^60^ Croatia,^72^ England,^34^ France,^36^ Malta,^42^ and Turkey^33^); 3 in Australia^37,38,58^; and 1 in Africa (Sudan).^53^ ACs were prescribed mainly for AF, VTE treatment, and VTE prophylaxis in patients undergoing various types of surgeries (see Supplementary material online, *Table S1*). Eleven studies^34,36,38,41,42,55–57,60,61,71^ included patients prescribed DOACs or warfarin, 10 studies^53,54,58,63,65,66,68–70,72^ warfarin alone, 6 studies^35,39,62,64,73,74^ DOACs, 4 studies^32,37,40,43^ parenteral ACs, and 4 studies^33,44,59,67^ any antithrombotic (including AC and/or antiplatelet) medications (used to prevent post-surgical VTE in patients who underwent surgery and stroke and other cardiac complications in those with AF and ischaemic heart disease).

The types of pharmacist-led interventions most commonly used were patient education or counselling,^32–34,37,38,41,53–55,57,61–63,69,72–74^ medication chart or patient review,^34,35,37,39,41,58,59,73,74^ bleeding, stroke, or VTE risk assessment,^34,40,43,56,59^ the development and implementation of pharmacist-driven protocols or guidelines,^32,39,40,42,44,56,60,62,73^ medication reconciliation,^56,59^ staff training,^36,56^ and providing alternative AC therapy or dose recommendations.^33,39,59,63,69^ Their impact in improving the appropriateness of AC therapy was examined in 14 studies.^32–44,60^ Their impact on clinical outcomes was addressed as follows: bleeding in 19 studies,^33,43,53–58,61,62,64–69,71,72,74^ thromboembolic events in 14 studies,^33,43,56–59,63,64,67–69,71,73,74^ readmission or hospitalization in 10 studies,^41,53,55,56,58,63,68–70,73^ and mortality in 6 studies^43,55,58,67,71,74^ (*Table 1*).

**Table 1:** Characteristics of the included studies in the review

Study, year and country	Study design and settings	Comparison groups	Sample size	Follow-up time (months)	Drugs used	Specific intervention(s) given	Key findings (control vs. intervention)
Falamić *et al.* 2019,^72^ Croatia	RCT, Community pharmacy	Control group Intervention group	66 65	6	Warfarin	• Patient education • Optimizing warfarin dose and contacting physicians to avoid drug interactions	• ↓ Bleeding (from 85% to 29%; *P* < 0.05)
Karaoui *et al*. 2021,^55^ Lebanon	RCT, Hospital	Standard of care Pharmacist-counsel	100 100	1	Apixaban Dabigatran Rivaroxaban VKA	• Counselling and education of patients	• ↓ Bleeding (from 17% to 14%) • ↔ Readmission rates (7% vs. 7%) • ↓ Mortality (from 4% to 2%)
Lakshmi *et al.* 2013,^61^ India	RCT, Hospital	Control group Intervention group	40 40	6	OACs	• Patient counselling • Providing patient information booklets	• ↓ Bleeding (from 85% to 60%)
Liang *et al.* 2020,^63^ China	RCT, Hospital	UC PEFS	75 77	43	Warfarin	• Patient education • Providing dose recommendations	• ↓ TE events (9.3% vs. 6.5%; *P* > 0.05) • ↔ Hospitalization (12% vs. 11.7%; *P* > 0.05)
Liu *et al*. 2022,^57^ China	RCT, Hospital	Control group Intervention group	64 61	6	Warfarin Rivaroxaban	• Medication guidance and monitoring • Patient education and other pharmaceutical care services	• ↓ Bleeding (from 43.8% to 13.1%; *P* < 0.05) • ↓ Thrombosis events (from 7.8% to 0%; *P* < 0.05)
Ahmed *et al.* 2017,^53^ Sudan	Quasi-experimental, Hospital	Before intervention After intervention	135 135	12	Warfarin	• Patient education and providing written information • Dose adjustments	• ↓ Bleeding (from 39.2% to 27.4%; *P* < 0.05) • ↓ Hospitalization (from 10.4% to 3.7%; *P* < 0.001)
Chong *et al.* 2021,^37^ Australia	Quasi-experimental, Hospital	Pre-stewardship Post-stewardship	400 411	6	Enoxaparin Heparin Others	• Education for medical officers • Chart auditing • Gamification and health promotion • Clinician performance feedback	• ↑ AC prescription appropriateness (from 78% to 88%; *P* = 0.004)
Gauci *et al.* 2019,^42^ Malta	Quasi-experimental, Hospital	Pre-implementation of MAT-AF Post-implementation of MAT-AF	150 150	7	Warfarin DOAC	• Implementing MAT-AF as a clinical tool • Documentation	• ↑ AC therapy appropriateness (from 70% to 88%)
Hyland *et al.*^a^ 2020,^59^ USA	Quasi-experimental, Hospital	Pre-implementation Post-implementation	694 533	6	Not reported	• Patient review and VTE risk stratification • Recommending TP medication selection, dose, and duration • Managing perioperative chronic antithrombotic therapies • Comprehensive discharge medication reconciliation	• ↓ Postoperative readmission rate (from 4.8% to 1.3%; *P* = 0.002) • ↓ VTE (from 0.6% to 0.0%; *P* = 0.13)
Khalil *et al*. 2021,^38^ Australia	Quasi-experimental, Hospital	Pre-intervention Post-intervention	61 65	7	Apixaban Rivaroxaban Dabigatran Warfarin	• Patient education • Providing a written AF brochure that contains treatment options	• ↑ AC therapy appropriateness (from 36% to 92%; *P* < 0.001)
Kiracı *et al*.^a^ 2023,^33^ Turkey	Quasi-experimental, Hospital	Pre-education CP intervention	340 191	2	Any TP	• Providing education to surgeons and residents • Providing optimal TP recommendations to prescribers	• ↑ TP appropriateness (from 46.8% to 61.3%; *P* = 0.001) • ↓ VTE (from 1.2% to 0%) • ↓ Bleeding (from 1.8% to 1.0%)
Lachuer *et al.* 2021,^36^ France	Quasi-experimental, Hospital	Pre-intervention Post-intervention	58 89	2.4	VKA DOAC	• Providing training for physicians	• ↑ Prescribing practices of VKA (from 74.4% to 77.9%) and DOAC (from 74% to 82.2%) • ↑ OAC prescription appropriateness (from 74.1% to 79.8%)
Lee *et al.* 2013,^62^ USA	Quasi-experimental, Hospital	UC Pharmacist-managed anticoagulation clinic	48 20	3	Dabigatran	• Patient education • Patient follow-up at 2 weeks, 1 and 3 months by means of telephone or face-to-face visits • Implementing pharmacy benefits management services guidelines	• ↔ Bleeding (4.2% vs. 10%; *P* = 0.148)
Miele *et al.* 2017,^39^ USA	Quasi-experimental, Hospital	Pre-intervention Post-intervention	50 85	3	Apixaban Dabigatran Rivaroxaban Edoxaban	• Developing and implementing pharmacist-driven protocol • Patient profile reviews • Providing an appropriate medication alternative to the prescriber	• ↑ DOAC prescription appropriateness (from 60% to 70.6%)
Quintens *et al.* 2022,^60^ Belgium	Quasi-experimental, Hospital	Pre-implementation Post-implementation	466 485	24	Enoxaparin Apixaban Rivaroxaban Edoxaban Dabigatran VKA	• Implementing a pharmacist-led CMA intervention	• ↓ AC-related residual potentially inappropriate prescriptions (from 74.9 to 22.5%) • ↓ Median proportion of residual PIPs (from 78.5% to 18.2%)
Sarika *et al.* 2021,^32^ India	Quasi-experimental, Hospital	Pre-intervention Post-intervention	45 45	5	Heparin Enoxaparin Fondaparinux	• Implementing ACCP guideline • Patient counselling	• ↑ Appropriateness of AC therapy (from 28.9% to 62.22%)
Shang *et al*. 2021,^56^ China	Quasi-experimental, Hospital	Pre-intervention Post-intervention	240 337	18	LMWH VKA Rivaroxaban	• Thrombosis and bleeding risk assessments • Consulting with physicians to formulate antithrombotic treatment protocols • Optimizing perioperative medication regimens • Evaluating and optimizing the feasibility of discharge prescriptions • Following up the thrombosis of TJA patients in the first and third months after surgery	• ↓ Deep vein thrombosis incidence (from 3.33% to 1.78%; *P* > 0.05) • ↔ Minor bleeding (6.67% vs. 5.93%; *P* = 0.720) • Improvement in TP use, administration timing, and treatment course at post-intervention (*P* < 0.001)
Sharma *et al.* 2024,^34^ England	Quasi-experimental, General practices	Pre-intervention Post-intervention	470 498	14	Apixaban Rivaroxaban Edoxaban Dabigatran Warfarin	• Medication review • Optimizing AF treatment • Patient counselling • Bleed risk assessment	• OAC prescriptions appropriateness (from 77% to 93.8%) • Successful transition from VKA to DOACs in 25.71% of patients
Tyedin *et al.* 2020,^58^ Australia	Quasi-experimental, Hospital	Pre-intervention Post-intervention	130 108	5	Warfarin	• Medication review • Proactively charting and monitoring • Ordering INRs using electronic prescribing software, following discussion with doctors	• ↓ Re-hospitalization (from 3.1% to 0%; *P* = 0.18) • ↓ Mortality (from 4.6% to 0%)
Yap *et al*. 2019,^40^ Malaysia	Quasi-experimental, Hospital	Pre-intervention Post-intervention	142 144	5	Fondaparinux Enoxaparin Heparin	• Developing and implementing a pharmacist-driven DVT Risk Alert Tool • DVT and bleeding risk assessment	• ↑ DVT prophylaxis appropriateness (from 14.3% to 31.3%; *P* < 0.05)
Wu *et al*. 2022,^54^ Taiwan	Quasi-experimental, Hospital	Conventional Pharmacist-managed group	39 33	24	Warfarin	• Patient and caregiver education • Developing a monitoring plan for INR and ADRs • Managing drug–drug and drug–food interactions • Participating in the ward check rounds •	• ↓ Major bleeding (1 vs. 0, in conventional vs. pharmacist-managed group) •
An *et al.* 2017,^65^ Japan	Retrospective cohort, Hospital	UC group Intervention group	32 25	28	Warfarin	• Monitoring of bleeding and PT-INR • Proposing to physicians to change the dose of warfarin when appropriate • Checking interaction of warfarin and supplements or foods	• ↓ Major bleeding (from 12.5% to 8%) • ↔ Minor bleeding (28% vs. 28.1%)
Ashjian *et al.* 2017,^35^ USA	Retrospective cohort, Health system data	UC Pharmacist-led DOAC	129 129	16	Dabigatran Rivaroxaban Apixaban	• Medication review	• ↑ DOAC appropriateness (from 81.1% to 93.7%; *P* = 0.016)
Bakey *et al.* 2022,^41^ USA	Retrospective cohort, Hospital	No pharmacist involvement Pharmacist involvement	44 14	12	Apixaban Rivaroxaban Warfarin Enoxaparin	• Patient counselling • Chart review • Evaluating OAC options • Providing additional written discharge instructions to patients	• ↑ AC appropriateness (from 70.5% to 92.9%; *P* = 0.046) • ↓ Readmission rates (from 20.5% to 7.1%; *P* = 0.424)
Derington *et al*. 2023,^71^ USA	Retrospective cohort, Hospital	UC AMS	6182 4939	39	Dabigatran Apixaban Rivaroxaban Warfarin	• Managing AC therapy • Evaluating DOAC appropriateness, dose-indication match, potential drug–drug interactions, needed laboratory measurements, and dose adjustments • Patient education • Ordering relevant laboratory tests	• ↔ Net clinical outcomes (major bleeding, TE, and mortality) (hazard ratio: 0.9, 95% CI: 0.7–1.0) • ↑ Major bleeding in patients with DOACs (hazard ratio: 1.5, 95% CI: 1.0–2.3) • ↓ Mortality in patients with DOAC (hazard ratio: 0.7, 95% CI: 0.6–0.9) • ↔ TE events (1.3% vs. 1.9%) • ↓ Mortality (from 13.3% to 11.6%)
DiRenzo *et al.* 2018,^73^ USA	Prospective cohort, Clot clinic	PCP Pharmacist-managed clinic	17 17	6	Rivaroxaban	• Patient counselling • Using the American College of Chest Physicians Guidelines (CHEST) • Reviewing patient profile • Identifying VTE symptoms and performing a physical examination	• ↔ TE events (6% vs. 6%; *P* = 1.000) • ↔ Hospitalization (6% vs. 6%; *P* = 1.000)
Han *et al.*^a^ 2021,^44^ USA	Retrospective cohort, Academic health centre	Physician led CP led	138 553	14	Warfarin Apixaban Rivaroxaban Dabigatran Antiplatelet	• Implementing the best practice alert in addition to their pharmacotherapy training	• ↑ Appropriateness of OAC discontinuation during pre-procedure (from 91% to 97.4%; *P* = 0.001) • ↑ Appropriateness of bridging therapy (from 50% to 72.2%; *P* = 0.39)
Jones *et al.* 2020,^74^ USA	Retrospective cohort, Healthcare system data	Non-AMS group AMS	370 90	42	Apixaban Rivaroxaban Dabigatran	• Patient education followed by phone calls • Chart reviews	• ↑ Composite outcomes (bleeding, TE, mortality) (from 13.5% to 18.9%; *P* = 0.29) • ↑ Bleeding (from 10.5% to 18.9%; *P* = 0.03) • ↓ TE events (from 1.1% to 0%) • ↓ Mortality (from 1.9% to 0%)
Kose *et al.* 2018,^66^ Japan	Retrospective cohort, Hospital	UC Intervention group	23 16	16	Warfarin	• Monitoring of bleeding and PT-INR • Checking interaction of warfarin and supplements or foods • Advising physicians to adjust the dose of warfarin as needed	• ↔ Major bleeding (17.4% vs. 18.8%) • ↓ Minor bleeding (from 43.5% to 31.3%)
Kurimura *et al.*^a^ 2023,^67^ Japan	Retrospective cohort, Hospital	Non-intervention group Intervention group	264 132	48	Warfarin Apixaban Rivaroxaban Dabigatran Edoxaban Antiplatelets	• Checking all prescription drugs from the hospital and other healthcare facilities • Pharmaceutical advice and suggesting prescription changes to physicians • Assessing adherence, adverse events, and discontinuation of unnecessary medications	• ↓ Bleeding (from 28.4% to 17.4%; *P* = 0.019) • ↓ TE (from 14% to 6.8%; *P* = 0.44) • ↑ Mortality (from 9% to 13.6%; *P* = 0.17)
Li *et al*. 2020,^64^ China	Prospective cohort, Hospital	UC PEFS	202 179	6	Rivaroxaban	• Observing potential interaction with rivaroxaban and managing bleeding and embolic complications • Evaluating patient medication adherence • Distributing paper-based medication education materials • Pharmacists kept in touch with the patients through WeChat or telephone weekly	• ↓ Bleeding (from 26.7% to 16.2%) • ↔ TE (9.4% vs. 7.8%; *P* = 0.675)
Manzoor *et al*. 2018,^70^ USA	Retrospective cohort, Hospital	NMAC PMAC	100 100	15	Warfarin	• Monitoring and managing patients receiving warfarin therapy	• ↑ Hospitalization or ED visits for patients in the NMAC group [eight times higher (OR: 7.68, 95% CI: 1.1–55.9, *P* < 0.05)]
Noor *et al.* 2021,^69^ Saudi Arabia	Retrospective cohort, Hospital	HMAC PMAC	124 104	24	Warfarin	• Patient counselling • Assessing INRs, warfarin therapy • Assessing ADRs, drug–drug interactions, or drug–food interactions • Documenting all therapeutic recommendations and prescribing a new warfarin prescription	• ↔ Bleeding (12.5% vs. 12.1%) • ↓ TE events (5.7% to 4%) • ↓ Hospitalization rates (from 9.6% to 4%)
Tarasiuk *et al*. 2018,^68^ USA	Retrospective cohort, Hospital	Nurse-managed group Pharmacist-managed group	240 228	12	Warfarin	• Managing patient dosing and monitoring autonomously utilizing clinical judgement	• ↓ Hospitalizations (OR: 0.29, *P* < 0.001) • ↓ Bleeding (from 2.1% to 1.3%) • ↓ TE (from 2.1% to 0.4%) • ↔ ED visits (39.2% vs. 43%; *P* = 0.402)
Zhang *et al*.^b^ 2023,^43^ China	Retrospective cohort, Hospital	Control group CP services group	162 176	21	Heparin	• Assessing VTE and bleeding risk on admission and giving VTE prophylaxis recommendations • Providing medication consultation to physicians, nurses, and patients	• ↑ TP appropriateness (from 59% to 84%) • ↓ TE events (from 17% to 9%; *P* = 0.037) • ↓ Bleeding (from 11% to 5%; *P* = 0.042) • ↓ Mortality (from 28% to 14%; *P* = 0.001)

Abbreviations: AC, anticoagulant; ACCP, American College of Clinical Pharmacy; ADR, adverse drug reaction; AF, atrial fibrillation; AMS, anticoagulation management service; CI, confidence interval; CMA, Check of Medication Appropriateness; CP, clinical pharmacist; DOAC, direct-acting oral anticoagulant; ED, emergency department; HMAC, haematologist-managed anticoagulation clinic; INR, international normalized ratio; LMWH, low-molecular-weight heparin; MAT-AF, medication assessment tool of atrial fibrillation; MUR, medication use review; NMAC, nurse-managed anticoagulation clinic; OAC, oral anticoagulant; OR, odds ratio; PEFS, pharmacist-led education and follow-up service; PMAC, pharmacist-managed anticoagulation clinic; PT-INR, prothrombin time–international normalized ratio; RCT, randomized controlled trial; TE, thromboembolic; TJA, total joint arthroplasty; TP, thromboprophylaxis; UC, usual care; VKA, vitamin K antagonist; VTE, venous thromboembolism.

^a^Included patients treated with antithrombotic medications (anticoagulants and/or antiplatelets).

^b^Included patients aged ≥16 years.

### Studies quality assessment

Based on JBI metrics, of the RCT studies, one^63^ was judged to be of high quality and four studies^55,57,61,72^ were rated as moderate quality (see Supplementary material online, *Table S2*). From the quasi-experimental studies, 14 studies^32–34,36–40,42,53,56,59,60,62^ were rated as high quality and 2 studies^54,58^ were judged to be of moderate quality (see Supplementary material online, *Table S3*). Most cohort studies^35,41,43,44,64–70,73,74^ were assessed as high quality (see Supplementary material online, *Table S4*); only the study by Derington *et al*.^71^ was judged to be of moderate quality. Blinding was a primary concern in RCTs, as either blinding status was unclear or participants, intervention providers, and outcome assessors were not blinded in four out of five studies.^55,57,61,72^ Quasi-experimental studies lacked a control group and showed baseline variations, while cohort studies were limited in addressing strategies to deal with confounding factors.

## Meta-analysis

### Appropriateness of anticoagulant therapy

**Figure 2:**
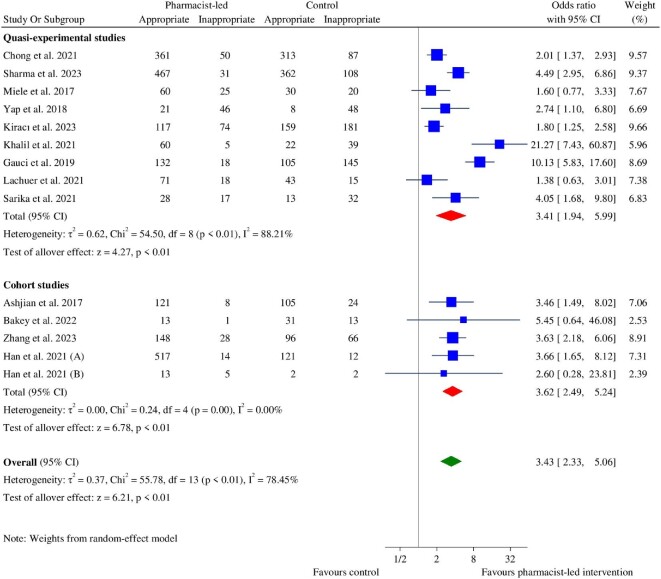
Forest plot of the appropriateness of anticoagulant therapy between the pharmacist-led intervention group and the usual care group.

Fourteen studies, 10 quasi-experimental studies,^32–34,36–40,42,60^ and 4 cohort studies^35,41,43,44^ reported the appropriateness of AC therapy. All studies except that by Quintens *et al*.,^60^ which reported the proportion of potentially inappropriate prescriptions that remained uncorrected after 48 h, were included in the meta-analysis. The meta-analysis (*Figure 2*) revealed an overall significant effect in favour of the pharmacist-led intervention on the appropriateness of AC therapy (OR: 3.43, 95% CI: 2.33–5.06, *P* < 0.01). This remained significant when subgroup analysis was performed based on study design: quasi-experimental (OR: 3.41, 95% CI: 1.94–5.99, *P* < 0.01) and cohort studies (OR: 3.62, 95% CI: 2.49–5.24, *P* < 0.01) (*Figure 2*). It also remained unchanged with subgroup analyses by medication types prescribed: all ACs (OR: 2.07, 95% CI: 1.42–3.01, *P* < 0.01), antithrombotics (including AC and/or antiplatelet) (OR: 2.32, 95% CI: 1.29–4.16, *P* < 0.01), DOACs (OR: 2.29, 95% CI: 1.08–4.85, *P* = 0.03), OACs (OR: 5.88, 95% CI: 1.96–17.64, *P* < 0.01), and parenteral ACs (OR: 3.52, 95% CI: 2.36–5.24, *P* < 0.01) (see Supplementary material online, *Figure S1, Panel A*). In the Quintens *et al*.^60^ study, the proportion of potentially inappropriate prescriptions that remained uncorrected after 48 h decreased from 78.5% to 18.2% by the pharmacist-led intervention.

High heterogeneity was observed among the included studies (χ^2^ = 55.78, *P* < 0.01, *I*^2^ = 78.45%), which was verified in quasi-experimental studies (χ^2^ = 54.50, *P* < 0.01, *I*^2^ = 88.21%) (*Figure 2*), and studies in Australia (χ^2^ = 17.14, *P* < 0.01, *I*^2^ = 94.17%) and Europe (χ^2^ = 33.41, *P* < 0.01, *I*^2^ = 92.02%) (see Supplementary material online, *Figure S1, Panel B*). The leave-one-out analyses revealed that Gauci *et al.*^42^ contributed the highest heterogeneity, and its exclusion decreased *I*^2^ from 78.45% to 70.15% (see Supplementary material online, *Figure S2, Panel A*). Furthermore, excluding the Australian study by Khalil *et al*.^38^ decreased *I*^2^ to 49% (see Supplementary material online, *Figure S2, Panel B*). The impact of pharmacist-led interventions in improving AC therapy appropriateness remained significant in the leave-one-out sensitivity analyses (see Supplementary material online, *Figure S3*). The overall OR did not change by more than 0.38 points [ranging from 3.05 (95% CI: 2.14–4.34) to 3.68 (95% CI: 2.45–5.53)]. We did not find publication bias by visual analysis of the funnel plot and Egger's test (*P* = 0.50).

### Bleeding events

**Figure 3:**
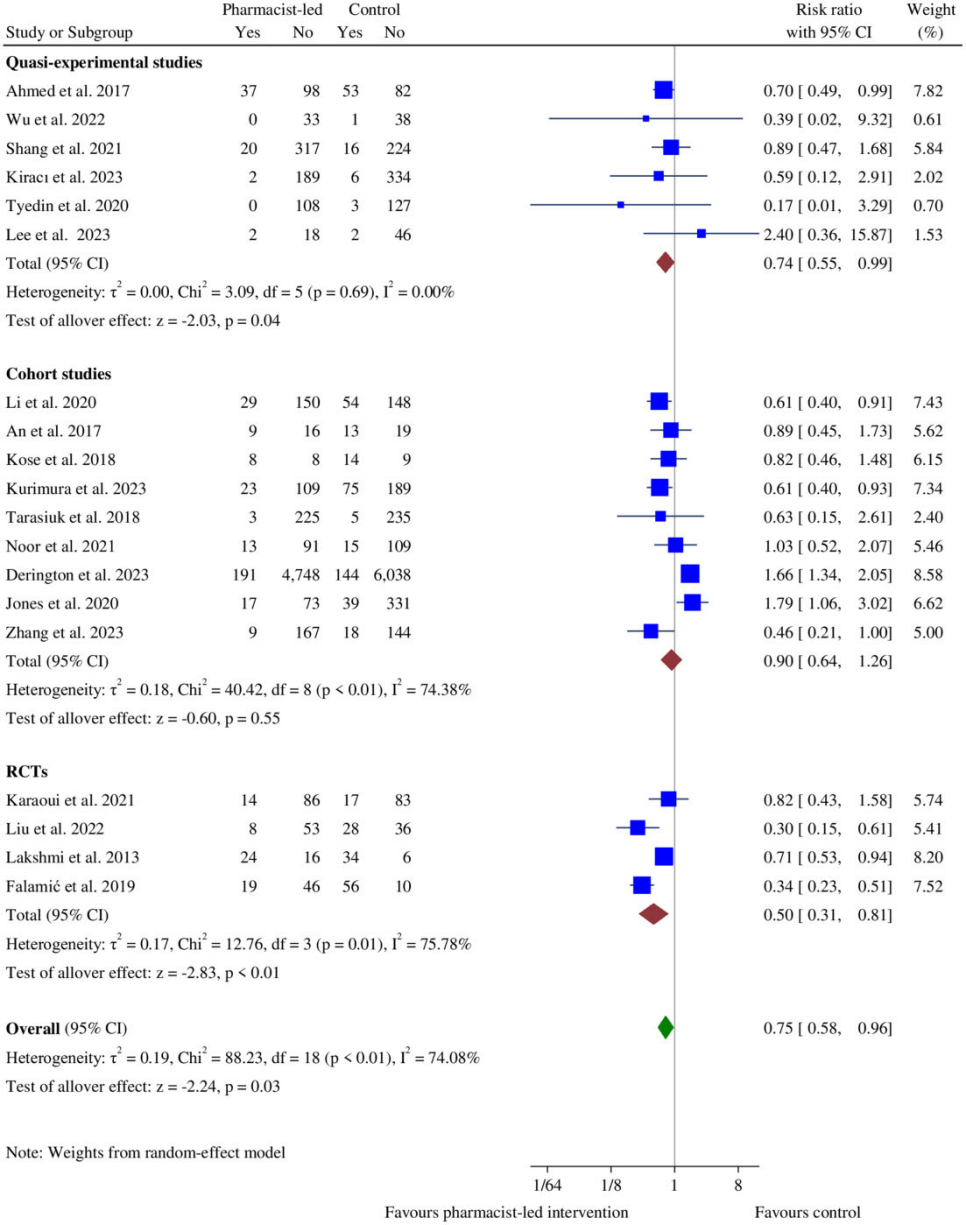
Forest plot of bleeding event between the pharmacist-led intervention group and the usual care group.

The pooled analysis of nine cohort studies,^43,64–69,71,74^ six quasi-experimental studies,^33,53,54,56,58,62^ and four RCTs^55,57,61,72^ showed that the pharmacist-led interventions significantly decreased the risk of total bleeding events (RR: 0.75, 95% CI: 0.58–0.96, *P* = 0.03) (*Figure 3*). In the subgroup analyses, the bleeding risk remained significantly decreased in quasi-experimental (RR: 0.74, 95% CI: 0.55–0.99, *P* = 0.04) and RCT (RR: 0.50, 95% CI: 0.31–0.81, *P* < 0.01) studies, but not in cohort studies (RR: 0.90, 95% CI: 0.64–1.26, *P* = 0.55) (*Figure 3*). Based on medication types, pharmacist-led interventions significantly decreased bleeding risk for patients taking warfarin (RR: 0.65, 95% CI: 0.45–0.94, *P* = 0.02) and antithrombotic medications (RR: 0.61, 95% CI: 0.41–0.92, *P* = 0.02), but not for those on DOACs (RR: 1.17, 95% CI: 0.48–2.82, *P* = 0.73) or OACs (RR: 0.77, 95% CI: 0.39–1.52, *P* = 0.45) (see Supplementary material online, *Figure S4, Panel A*). Additionally, the bleeding risk was reduced in the pharmacist-led intervention groups in both Asian (RR: 0.68, 95% CI: 0.58–0.79, *P* < 0.01) and European studies (RR: 0.36, 95% CI: 0.24–0.52, *P* < 0.01) (see Supplementary material online, *Figure S4, Panel B*). Substantial heterogeneity (χ^2^ = 88.23, *P* < 0.01, *I*^2^ = 74.08%) was found among the included studies, specifically in cohort studies (χ^2^ = 40.42, *P* < 0.01, *I*^2^ = 74.38%) and RCTs (χ^2^ = 12.76, *P* = 0.01, *I*^2^ = 75.78%). Using leave-one-out analyses, we found that the study by Derington *et al*.^71^ contributed the highest heterogeneity. Its exclusion decreased *I*^2^ from 74.08% to 59.39% (see Supplementary material online, *Figure S5*). No publication bias was indicated by Egger's test (*P* = 0.52) or visual inspection of the funnel plot. In the leave-one-out sensitivity analyses, the overall RR of bleeding ranged from 0.69 (95% CI: 0.55–0.87) to 0.80 (95% CI: 0.63–1.02) and remained significant at all times except when the studies by Falamić *et al*.^72^ and Liu *et al*.^57^ were excluded (see Supplementary material online, *Figure S6*).

Two RCTs,^55,61^ three quasi-experimental studies,^33,54,62^ and six cohort studies^57,65,66,69,71,74^ reported major bleeding events. Their pooled estimate indicated no significant difference in decreasing major bleeding between the pharmacist-led interventions and UC (RR: 0.99, 95% CI: 0.59–1.66, *P* = 0.96). Furthermore, minor bleeding events from two quasi-experimental studies,^56,62^ three RCTs,^55,57,61^ and four cohort studies^65,66,69,74^ showed no significant difference between the pharmacist-led intervention and control groups (RR: 0.84, 95% CI: 0.61–1.17, *P* = 0.31) (see Supplementary material online, *Figure S7*).

### Thromboembolic events

**Figure 4:**
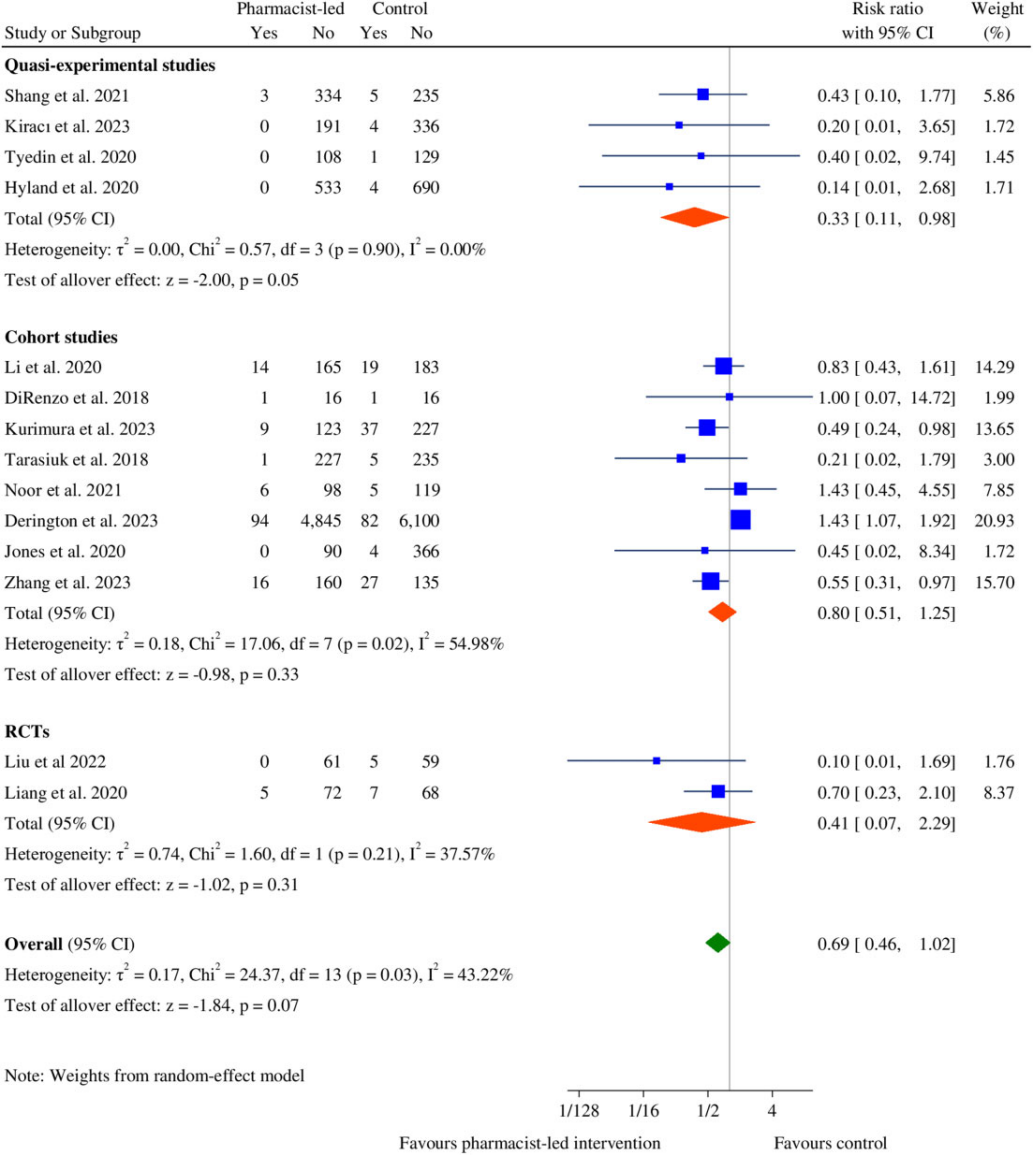
Forest plot of thromboembolic event between the pharmacist-led intervention group and the usual care group.

**Figure 5:**
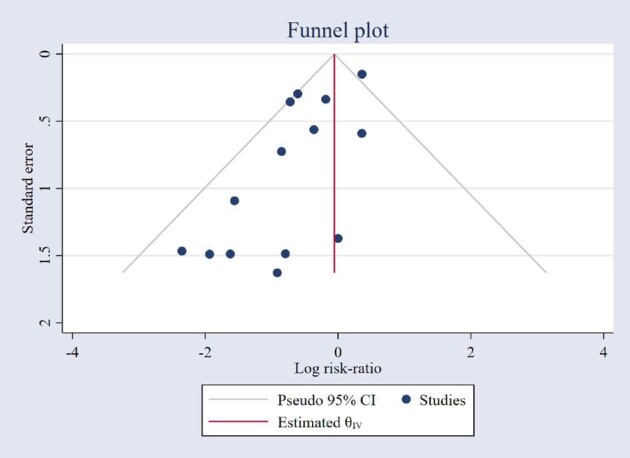
Funnel plot indicating publication bias in thromboembolic event analysis studies.

Fourteen studies (2 RCTs,^57,63^ 4 quasi-experimental studies,^33,56,58,59^ and 8 cohort studies^43,64,67–69,71,73,74^) reported thromboembolic events. The overall pooled result showed a non-significant decrease in the risk of thromboembolic events in the pharmacist-led intervention groups (RR: 0.69, 95% CI: 0.46–1.02, *P* = 0.07) (*Figure 4*). Using subgroup analyses, pharmacist-led interventions did not significantly decrease thromboembolic events in the cohort (RR: 0.80, 95% CI: 0.51–1.25, *P* = 0.33) and RCT (RR: 0.41, 95% CI: 0.07–2.29, *P* = 0.31) studies, but did in quasi-experimental studies (RR: 0.33, 95% CI: 0.11–0.98, *P* = 0.05) (*Figure 4*). Additional subgroup analysis indicated that interventions led by pharmacists did not significantly reduce thromboembolic events in American studies (RR: 0.69, 95% CI: 0.24–2.01, *P* = 0.49) or in patients receiving DOACs (RR: 0.82, 95% CI: 0.44–1.53, *P* = 0.53) and warfarin (RR: 0.78, 95% CI: 0.38–1.62, *P* = 0.51); however, a significant reduction was observed in Asian studies (RR: 0.63, 95% CI: 0.45–0.87, *P* < 0.01) (see Supplementary material online, *Figure S8*). Egger's test (*P* = 0.03) and the trim-and-fill method (see Supplementary material online, *Figure S9*) indicated publication bias, supported by visual analysis funnel plots (*Figure 5*).

Moderate heterogeneity (χ^2^ = 24.37, *P* = 0.03, *I*^2^ = 43.22%) was found among the included studies, specifically in cohort studies (χ^2^ = 17.06, *P* = 0.02, *I*^2^ = 54.98%). In the leave-one-out analyses, we identified that the study by Derington *et al.*^71^ significantly influenced the overall pooled estimate, increased heterogeneity among included studies, and introduced publication bias. Excluding this study, pharmacist-led interventions significantly decreased thromboembolic events (RR: 0.59, 95% CI: 0.43–0.81, *P* < 0.01) with no detected publication bias (see Supplementary material online, *Figure S10*). In the leave-one-out sensitivity analyses, the overall RR remained non-significant except when the study by Derington *et al*.^71^ or Noor *et al*.^69^ was excluded and ranged from 0.59 (95% CI: 0.43–0.81) to 0.73 (95% CI: 0.47–1.12) (see Supplementary material online, *Figure S11*).

### Mortality

**Figure 6:**
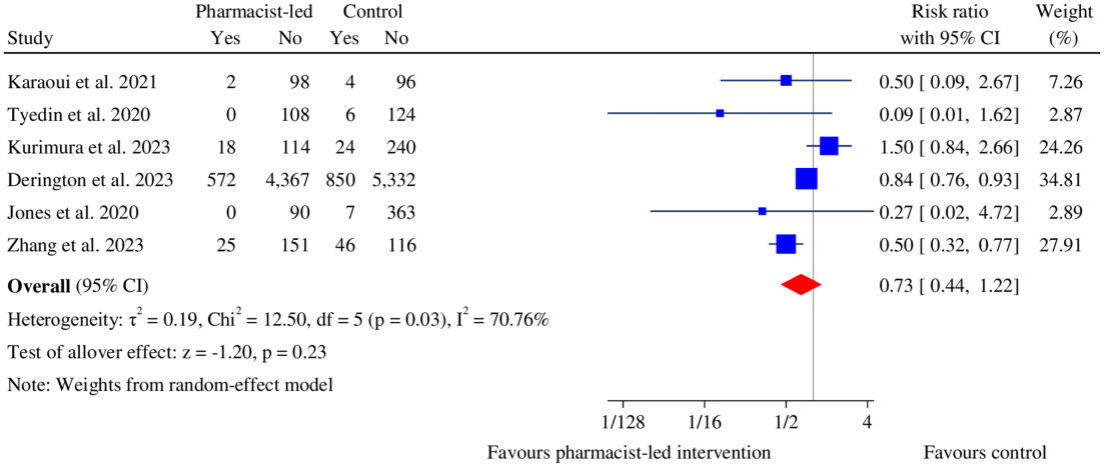
Forest plot of mortality between the pharmacist-led intervention group and the usual care group.

Patient deaths were reported in one quasi-experimental study,^58^ one RCT,^55^ and four cohort studies.^43,67,71,74^ There was no significant reduction of all-cause mortality in the pharmacist-led intervention groups (RR: 0.73, 95% CI: 0.44–1.22, *P* = 0.23), with substantial heterogeneity (χ^2^ = 12.50, *P* = 0.03, *I*^2^ = 70.76%) among the studies (*Figure 6*).

### Hospitalization or readmission

**Figure 7:**
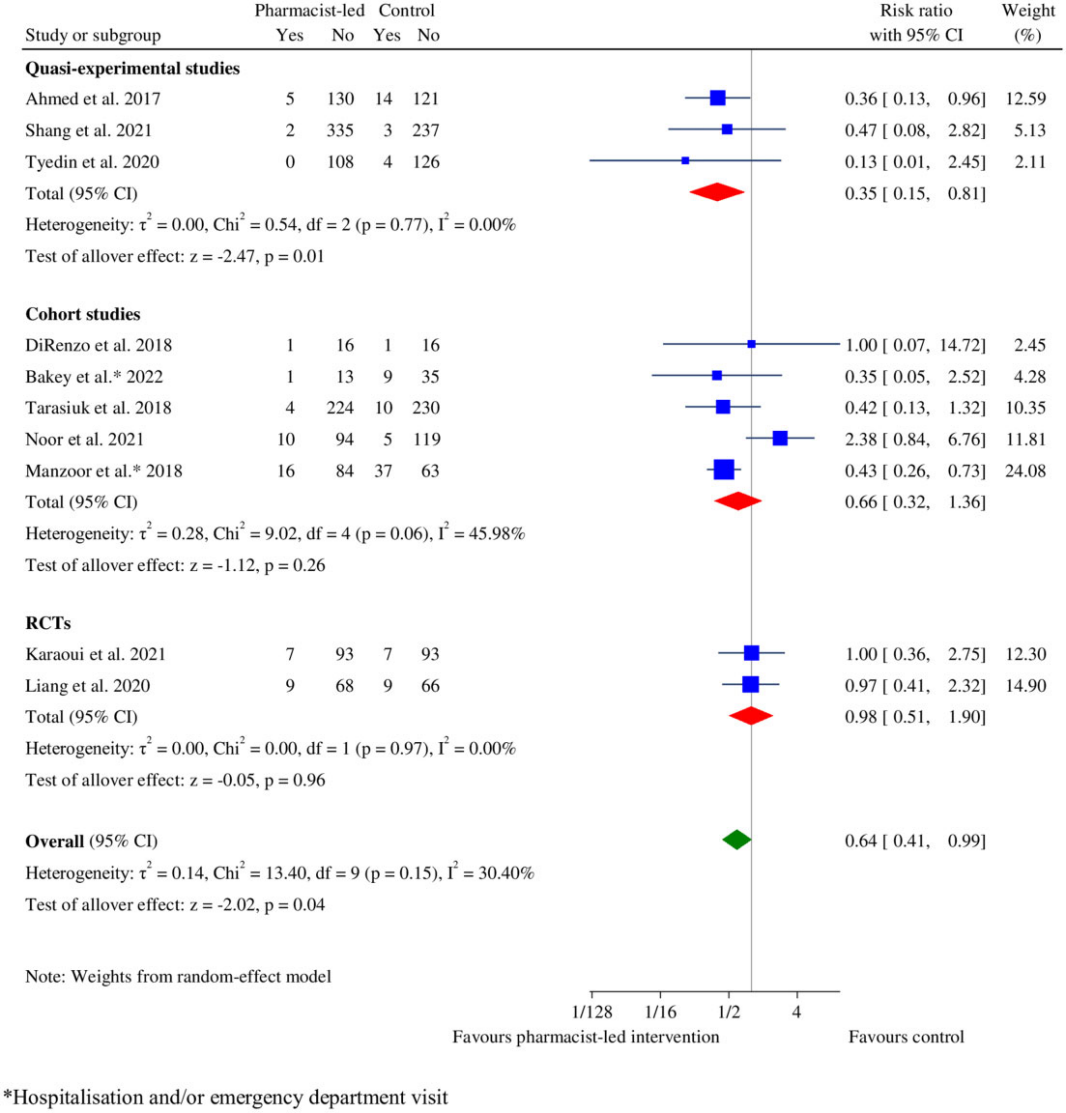
Forest plot of hospitalization between the pharmacist-led intervention group and the usual care group.

Hospitalization or readmission was reported in two RCTs,^55,63^ three quasi-experimental studies,^53,56,58^ and five cohort studies.^41,68–70,73^ The pharmacist-led intervention groups showed a significant reduction (RR: 0.64, 95% CI: 0.41–0.99, *P* = 0.04) in hospitalization or readmission, with low heterogeneity (χ^2^ = 13.40, *P* = 0.15, *I*^2^ = 30.40%) (*Figure 7*). Pharmacist-led interventions significantly decreased the risk of hospitalization or readmission in quasi-experimental studies (RR: 0.35, 95% CI: 0.15–0.81, *P* = 0.01), but not in RCTs (RR: 0.98, 95% CI: 0.51–1.90, *P* = 0.96) and cohort studies (RR: 0.66, 95% CI: 0.32–1.36, *P* = 0.26) (*Figure 7*). The overall RR ranged from 0.52 (95% CI: 0.37–0.73) to 0.72 (95% CI: 0.44–1.19) in sensitivity analyses, and when four studies (Karaoui *et al*.,^55^ Liang *et al*.,^63^ DiRenzo *et al*.,^73^ and Noor *et al*.^69^) were sequentially excluded, they remained consistent with the overall RR of the main analysis (see Supplementary material online, *Figure S13*). No publication bias was indicated by Egger's test (*P* = 0.6876) and visual analysis of the funnel plot.

## Discussion

AC therapies are frequently underused and inappropriately prescribed for AF and VTE, despite the existence of evidence-based guidelines.^17,19^ To our knowledge, this review is the first to assess the impact of pharmacist-led interventions on improving the appropriateness of AC therapy. Pharmacists optimize AC therapy through various integrated care management interventions.^75^ This review highlights predominant pharmacist-led interventions: patient education or counselling, medication charts or patient reviews, and developing and implementing pharmacist-driven protocols or guidelines. Patients receiving pharmacist-led care were three times more likely to be on appropriate AC therapy. This finding was consistent across study design, country, indication, and AC medication type.

Regarding the impact of pharmacist-led interventions on clinical outcomes, the findings of this meta-analysis were mixed. Prior research has identified that AC therapy is associated with preventable ADEs in hospitalized patients, most notable of which is bleeding.^10,11,76^ A previous review of eight RCTs and nine cohort studies^27^ found that the risk of bleeding was reduced by 57% in pharmacist-managed anticoagulation clinics compared with other models in patients prescribed warfarin (RR: 0.43, *P* = 0.006). Our meta-analysis, which focused on the post-DOAC era, found that pharmacist-led interventions reduced the risk of total bleeding events by 25%, with this being driven by a 50% reduction in bleeding in the subgroup analysis of the RCTs. Our subgroup analysis revealed that pharmacist-led interventions did not significantly reduce bleeding in DOAC studies. However, it did reduce bleeding by 35% in warfarin studies. Pharmacist-led interventions were more effective in Asian studies, which were mainly conducted on warfarin, compared with the North American studies, which mainly examined DOACs. The higher bleeding risk associated with warfarin, as compared with DOACs, could potentially be mitigated significantly through close monitoring by pharmacists in Asia involved in the intervention.

While there was also a significant 36% reduction in hospitalizations or readmissions with pharmacist-led interventions compared with UC, there were no significant differences in thromboembolic events. This might be due to the inclusion of the study by Derington *et al.*,^71^ a disproportionately large cohort study that had dissimilarities between groups at baseline that were not accounted for, as well as the non-restricted involvement of pharmacists in the UC DOAC model. Notably, when this cohort study was excluded from the meta-analysis in sensitivity analyses, pharmacist-led interventions significantly reduced thromboembolic events by 41% compared with UC (RR: 0.59, *P* < 0.01).

The non-significant difference in mortality observed with the pharmacist interventions in comparison with UC may be due to the low event rate. Specifically, only one RCT and one quasi-experimental study, each with a small number of patients and a short follow-up duration, could be included in this analysis. Detecting the effect of pharmacist-led interventions on this outcome requires large studies and extended follow-up periods.

The strength of our inferences relied on adhering to PRISMA guidelines for a rigorous systematic review. This included following a predesigned protocol to address the research question, employing a meticulous method for identifying relevant studies, rigorously assessing the methodological quality of the included studies, exploring sources of heterogeneity, and quantitatively summarizing the evidence. However, this review also has some limitations. Firstly, the studies included in our review showed significant statistical heterogeneity in AC therapy appropriateness and bleeding events, requiring caution in interpretation. Heterogeneity may have arisen from varying definitions of AC therapy appropriateness; some studies defined it based on guideline compliance, others on summaries of product characteristics, and some on specific local criteria. However, in our appropriateness analysis, consistent estimates across studies (irrespective of study design and country) suggest the absence of clinical heterogeneity, indicating the high generalizability of pharmacist-led interventions to various circumstances. Heterogeneity in the meta-analysis of bleeding was apparently due to one study.^71^ As seen in our meta-analysis, the outlier study might have affected the *I*^2^, which tends to increase when studies with very large sample sizes are included.^77^ Secondly, most of the studies were conducted at single centres (hospital settings) with varied interventions, and we could not confirm the most effective intervention and did not assess the cost-effectiveness of pharmacist-led interventions. Future research is needed to identify which pharmacist-led interventions are most effective and to determine their cost-effectiveness, including in community settings. Also, most studies were conducted in high-income countries, potentially limiting the generalizability of the findings to developing countries.

## Conclusion

This review has shown that pharmacist-led interventions improve AC therapy appropriateness and reduce total bleeding and hospitalizations or readmissions. However, the effect on thromboembolic events and mortality remains inconclusive. Given their proven benefits, policymakers and guideline developers should consider translating pharmacist-led interventions for optimizing AC therapy, particularly in hospitals. Further research is needed to evaluate pharmacist-led interventions’ cost-effectiveness and long-term sustainability.

## Supplementary Material

qcae045_Supplemental_Files

## Data Availability

All data are available in the manuscript or the supplemental files.

## References

[1] Umerah CO, Momodu II. Anticoagulation. Treasure Island, FL: StatPearls Publishing LLC; 2023. https://www.ncbi.nlm.nih.gov/books/NBK560651/ (Accessed 4 October 2023).

[2] Favaloro EJ, McCaughan GJB, Mohammed S, Pasalic L. Anticoagulation therapy in Australia. Ann Blood 2018;3:48.

[3] Doost A, Alasady M, Scott P. National Heart Foundation of Australia and the Cardiac Society of Australia and New Zealand: Australian Clinical Guidelines for the diagnosis and management of atrial fibrillation 2018. Heart Lung Circ 2019;28:e106–e1e7. 30935623 10.1016/j.hlc.2018.11.016

[4] Smith SN, Lanham M, Seagull FJ, Dorsch M, Errickson J, Barnes GD. Implementing pharmacist-prescriber collaboration to improve evidence-based anticoagulant use: a randomized trial. Implement Sci 2023;18:16. 37189171 10.1186/s13012-023-01273-4 PMC10184412

[5] Australian Institute of Health and Welfare (AIHW) . Heart, Stroke and Vascular Disease: Australian Facts. Atrial Fibrillation. Canberra: AIHW; 2020. https://www.aihw.gov.au/reports/heart-stroke-vascular-diseases/hsvd-facts/contents/all-heart-stroke-and-vascular-disease/atrial-fibrillation (Accessed 2 October 2023).

[6] Amaraneni A, Chippa V, Rettew AC. Anticoagulation Safety. Treasure Island, FL: StatPearls Publishing LLC; 2024. 30085567

[7] Coleman JJ, Pontefract SK. Adverse drug reactions. Clin Med 2016;16:481. 10.7861/clinmedicine.16-5-481 PMC6297296 27697815

[8] Frazer A, Rowland J, Mudge A, Barras M, Martin J, Donovan P. Systematic review of interventions to improve safety and quality of anticoagulant prescribing for therapeutic indications for hospital inpatients. Eur J Clin Pharmacol 2019;75:1645–1657. 31511939 10.1007/s00228-019-02752-8

[9] Palareti G, Antonucci E, Mastroiacovo D, Ageno W, Pengo V, Poli D et al. The American College of Chest Physician score to assess the risk of bleeding during anticoagulation in patients with venous thromboembolism. J Thromb Haemost 2018;16:1994–2002. 30059189 10.1111/jth.14253

[10] Schulman S, Beyth RJ, Kearon C, Levine MN. Hemorrhagic complications of anticoagulant and thrombolytic treatment: American College of Chest Physicians Evidence-Based Clinical Practice Guidelines (8th Edition). Chest 2008;133:257s–298s. 18574268 10.1378/chest.08-0674

[11] Little D, Chai-Adisaksopha C, Hillis C, Witt D, Monreal M, Crowther M et al. Resumption of anticoagulant therapy after anticoagulant-related gastrointestinal bleeding: a systematic review and meta-analysis. Thromb Res 2019;175:102–109. 30743134 10.1016/j.thromres.2019.01.020

[12] da Silva IRF, Frontera JA. Resumption of anticoagulation after intracranial hemorrhage. Curr Treat Options Neurol 2017;19:1–21. 28965189 10.1007/s11940-017-0477-y

[13] Zwicker J, Leaf RK, Carrier M. A meta-analysis of intracranial hemorrhage in patients with brain tumors receiving therapeutic anticoagulation. J Thromb Haemost 2016;14:1736–1740. 27306689 10.1111/jth.13387

[14] Shen N-N, Zhang C, Hang Y, Li Z, Kong L-C, Wang N et al. Real-world prevalence of direct oral anticoagulant off-label doses in atrial fibrillation: an epidemiological meta-analysis. Front Pharmacol 2021;12:581293. 34122056 10.3389/fphar.2021.581293 PMC8188240

[15] van der Horst S, van Rein N, van Mens T, Huisman M, Klok F. Inappropriate prescriptions of direct oral anticoagulants (DOACs) in hospitalized patients: a narrative review. Thromb Res 2023;231:135–140. 37005194 10.1016/j.thromres.2023.03.007

[16] Pritchett RV, Bem D, Turner GM, Thomas GN, Clarke JL, Fellows R et al. Improving the prescription of oral anticoagulants in atrial fibrillation: a systematic review. Thromb Haemostasis 2019;119:294–307. 30669165 10.1055/s-0038-1676835

[17] Kefale AT, Bezabhe WM, Peterson GM. Oral anticoagulant use in patients with atrial fibrillation at low risk of stroke and associated bleeding complications. J Clin Med 2023;12:6182. 37834830 10.3390/jcm12196182 PMC10573191

[18] Bezabhe WM, Bereznicki LR, Radford J, Wimmer BC, Curtain C, Salahudeen MS et al. Ten-year trends in the use of oral anticoagulants in Australian general practice patients with atrial fibrillation. Front Pharmacol 2021;12:586370. 33867975 10.3389/fphar.2021.586370 PMC8044929

[19] Steinberg BA, Gao H, Shrader P, Pieper K, Thomas L, Camm AJ et al. International trends in clinical characteristics and oral anticoagulation treatment for patients with atrial fibrillation: results from the GARFIELD-AF, ORBIT-AF I, and ORBIT-AF II registries. Am Heart J 2017;194:132–140. 29223431 10.1016/j.ahj.2017.08.011

[20] Ingram SJ, Kirkdale CL, Williams S, Hartley E, Wintle S, Sefton V et al. Moving anticoagulation initiation and monitoring services into the community: evaluation of the Brighton and Hove community pharmacy service. BMC Health Serv Res 2018;18:1–7. 29415718 10.1186/s12913-018-2901-8 PMC5803888

[21] Elewa H, AbdelSamad O, Elmubark A, Al-Taweel H, Mohamed A, Kheir N et al. The first pharmacist-managed anticoagulation clinic under a collaborative practice agreement in Qatar: clinical and patient-oriented outcomes. J Clin Pharm Ther 2016;41:403–408. 27144477 10.1111/jcpt.12400

[22] Eldooma I, Maatoug M, Yousif M. Outcomes of pharmacist-led pharmaceutical care interventions within community pharmacies: narrative review. Integr Pharm Res Pract 2023;12:113–126. 37216033 10.2147/IPRP.S408340 PMC10198268

[23] Sherwin E, Schaefer M, Huffmyer M, Naseman K, Davis GA, Schadler A et al. Evaluation of a pharmacist-led drive-up anticoagulation clinic during the coronavirus 2019 pandemic. J Am Pharm Assoc (Wash DC) 2023;63:151–157.e2.

[24] Alshaiban A, Alavudeen SS, Alshahrani I, Kardam AM, Alhasan IM, Alasiri SA et al. Impact of clinical pharmacist running anticoagulation clinic in Saudi Arabia. J Clin Med 2023;12:3887. 37373582 10.3390/jcm12123887 PMC10299558

[25] Lee T, Davis E, Kielly J. Clinical impact of a pharmacist-led inpatient anticoagulation service: a review of the literature. Integr Pharm Res Pract 2016;**5**:53–63. 29354540 10.2147/IPRP.S93312 PMC5741038

[26] Cheema E, Alhomoud FK, Kinsara ASA-D, Alsiddik J, Barnawi MH, Al-Muwallad MA et al. The impact of pharmacists-led medicines reconciliation on healthcare outcomes in secondary care: a systematic review and meta-analysis of randomized controlled trials. PLoS One 2018;13:e0193510. 29590146 10.1371/journal.pone.0193510 PMC5873985

[27] Hou K, Yang H, Ye Z, Wang Y, Liu L, Cui X. Effectiveness of pharmacist-led anticoagulation management on clinical outcomes: a systematic review and meta-analysis. J Pharm Pharm Sci 2017;20:378–396. 29145935 10.18433/J3SQ0B

[28] Zhou S, Sheng X, Xiang Q, Wang Z, Zhou Y, Cui Y. Comparing the effectiveness of pharmacist-managed warfarin anticoagulation with other models: a systematic review and meta-analysis. J Clin Pharm Ther 2016;41:602–611. 27677651 10.1111/jcpt.12438

[29] Shields AM, Lip GY. Choosing the right drug to fit the patient when selecting oral anticoagulation for stroke prevention in atrial fibrillation. J Intern Med 2015;278:1–18. 25758241 10.1111/joim.12360

[30] Kefale B, Peterson G, Bezabhe W. Pharmacist-led interventions and outcomes in patients with anticoagulant therapy. *PROSPERO* 2023; CRD42023487362. https://www.crd.york.ac.uk/prospero/display_record.php?ID=CRD42023487362 (Accessed 10 December 2023).

[31] Page MJ, Moher D, Bossuyt PM, Boutron I, Hoffmann TC, Mulrow CD et al. PRISMA 2020 explanation and elaboration: updated guidance and exemplars for reporting systematic reviews. BMJ; doi:10.1136/bmj.n160. Published online ahead of print 29 March 2021. PMC8005925 33781993

[32] Sarika A, Reghu A, Karattuthodi MS, Sreelatha ARP. Clinical pharmacist directed anticoagulation monitoring services: a prospective interventional study. İstanbul J Pharm 2021;51:291–298.

[33] Kiracı ZK, Yalçın N, Cennet Ö, Demirkan K, Yorgancı K. Education and clinical pharmacist-led management strategies for the risk and prophylaxis of venous thromboembolism in general surgery. Thromb J 2023;21:86. 37559115 10.1186/s12959-023-00530-2 PMC10413499

[34] Sharma R, Hasan SS, Gilkar IA, Hussain WF, Conway BR, Ghori MU. Pharmacist-led interventions in optimising the use of oral anticoagulants in atrial fibrillation patients in the general practice in England: a retrospective observational study. BJGP Open; doi:10.3399/BJGPO.2023.0113. Published online ahead of print 17 April 2024. PMC11300970 38097269

[35] Ashjian E, Kurtz B, Renner E, Yeshe R, Barnes GD. Evaluation of a pharmacist-led outpatient direct oral anticoagulant service. Am J Health Syst Pharm 2017;74:483–489. 28336758 10.2146/ajhp151026

[36] Lachuer C, Benzengli H, Do B, Rwabihama J-P, Leglise P, eds. Oral anticoagulants: interventional pharmaceutical study with reminder of good practices, and iatrogenic impact. Ann Pharm Franç 2021;79:409–417. 33516717 10.1016/j.pharma.2021.01.004

[37] Chong J, Curtain C, Gad F, Passam F, Soo G, Levy R et al. Development and implementation of venous thromboembolism stewardship across a hospital network. Int J Med Inform 2021;155:104575. 34560489 10.1016/j.ijmedinf.2021.104575

[38] Khalil V, Blackley S, Subramaniam A. Evaluation of a pharmacist-led shared decision-making in atrial fibrillation and patients’ satisfaction—a before and after pilot study. Ir J Med Sci 2021;190:819–824. 32808181 10.1007/s11845-020-02343-y

[39] Miele C, Taylor M, Shah A. Assessment of direct oral anticoagulant prescribing and monitoring pre-and post-implementation of a pharmacy protocol at a community teaching hospital. Hosp Pharm 2017;52:207–213. 28439135 10.1310/hpj5203-207 PMC5396988

[40] Yap DFS, Ng ZY, Wong CY, Saifuzzaman MM, Yang L. Appropriateness of deep vein thrombosis (DVT) prophylaxis use among medical inpatients: a DVT Risk Alert Tool (DRAT) study. Med J Malaysia 2019;74:45. 30846662

[41] Bakey KH, Nguyen C-TN. Impact of a pharmacist intervention in the emergency department on the appropriateness of direct oral anticoagulants prescribed in venous thromboembolism patients. J Pharm Pract 2022;35:599–605. 33736522 10.1177/08971900211000704

[42] Gauci M, Wirth F, Azzopardi LM, Serracino-Inglott A. Clinical pharmacist implementation of a medication assessment tool for long-term management of atrial fibrillation in older persons. Pharm Pract (Granada) 2019;17:1349. 31015870 10.18549/PharmPract.2019.1.1349 PMC6463411

[43] Zhang L, Wang Y, Zhang K, Li P, Qiao Y, Wang H et al. Impact of clinical pharmacist services on physicians’ guideline compliance and prognosis of patients for venous thromboembolism prophylaxis in ICU. Int J Clin Pharmacol Ther 2023;61:24–32. 36366968 10.5414/CP204289

[44] Han H, Chung G, Sippola E, Chen W, Morgan S, Renner E et al. Improving preprocedure antithrombotic management: implementation and sustainment of a best practice alert and pharmacist referral process. Res Pract Thromb Haemost 2021;5:e12558. 34296057 10.1002/rth2.12558 PMC8285271

[45] Tufanaru C, Munn Z, Aromataris E, Campbell J, Hopp L. Chapter 3: systematic reviews of effectiveness. In: Aromataris E, Munn Z, eds. JBI Manual for Evidence Synthesis. North Adelaide, Australia: JBI; 2020. 10.46658/JBIMES-20-04 (Accessed 10 November 2023).

[46] Moola S, Munn Z, Tufanaru C, Aromataris E, Sears K, Sfetcu R et al. Chapter 7: systematic reviews of etiology and risk. In: Aromataris E, Munn Z, eds. JBI Manual for Evidence Synthesis. North Adelaide, Australia: JBI; 2020. 10.46658/JBIMES-20-08 (Accessed 10 November 2023).

[47] Dijkshoorn AB, van Stralen HE, Sloots M, Schagen SB, Visser-Meily JM, Schepers VP. Prevalence of cognitive impairment and change in patients with breast cancer: a systematic review of longitudinal studies. Psychooncology 2021;30:635–648. 33533166 10.1002/pon.5623 PMC8248098

[48] Higgins JPT, Thomas J, Chandler J, Cumpston M, Li T, Page MJ, Welch VA, eds. Cochrane Handbook for Systematic Reviews of Interventions. Version 6.4. Cochrane; 2023. www.training.cochrane.org/handbook (Accessed 10 November 2023).

[49] Higgins JP, Thompson SG. Quantifying heterogeneity in a meta-analysis. Stat Med 2002;21:1539–1558. 12111919 10.1002/sim.1186

[50] Pei YF, Tian Q, Zhang L, Deng HW. Exploring the major sources and extent of heterogeneity in a genome-wide association meta-analysis. Ann Hum Genet 2016;80:113–122. 26686198 10.1111/ahg.12143 PMC4761279

[51] Duval S, Tweedie R. Trim and fill: a simple funnel-plot–based method of testing and adjusting for publication bias in meta-analysis. Biometrics 2000;56:455–463. 10877304 10.1111/j.0006-341x.2000.00455.x

[52] Sterne JA, Egger M, Smith GD. Investigating and dealing with publication and other biases in meta-analysis. BMJ 2001;323:101–105. 11451790 10.1136/bmj.323.7304.101 PMC1120714

[53] Ahmed NO, Osman B, Abdelhai YM, El-Hadiyah TMH. Impact of clinical pharmacist intervention in anticoagulation clinic in Sudan. Int J Clin Pharm 2017;39:769–773. 28508324 10.1007/s11096-017-0475-x

[54] Wu C-W, Wu C-C, Chen C-H, Lin S-Y, Hsu R-B, Huang C-F. The impact of pharmacist-managed service on warfarin therapy in patients after mechanical valve replacement. Int J Clin Pract 2022;2022:1617135. 35685594 10.1155/2022/1617135 PMC9159219

[55] Karaoui LR, Ramia E, Mansour H, Haddad N, Chamoun N. Impact of pharmacist-conducted anticoagulation patient education and telephone follow-up on transitions of care: a randomized controlled trial. BMC Health Serv Res 2021;21:1–12. 33593336 10.1186/s12913-021-06156-2 PMC7885504

[56] Shang J, Ning W, Gong J, Su D, Jia X, Wang Y. Impact of clinical pharmacist services on anticoagulation management of total joint arthroplasty: a retrospective observational study. J Clin Pharm Ther 2021;46:1301–1307. 33904165 10.1111/jcpt.13428

[57] Liu X, Xiao Q, Li Y, Hu G, Kun W, Xu W. Effect of post-discharge pharmacist-led follow-up on drug treatment in patients with deep venous thrombosis in primary hospitals in China. Pak J Pharm Sci 2022;35:785–791. 35791477

[58] Tyedin AE, Taylor SE, Than J, Al-Alawi R, O'Halloran E, Chau AH. Impact of proactive pharmacist-assisted warfarin management using an electronic medication management system in Australian hospitalised patients. J Pharm Pract Res 2020;50:144–151.

[59] Hyland SJ, Kramer BJ, Fada RA, Lucki MM. Clinical pharmacist service associated with improved outcomes and cost savings in total joint arthroplasty. J Arthroplasty 2020;35:2307–2317.e1. 32389406 10.1016/j.arth.2020.04.022

[60] Quintens C, Verhamme P, Vanassche T, Vandenbriele C, Van den Bosch B, Peetermans WE et al. Improving appropriate use of anticoagulants in hospitalised patients: a pharmacist-led Check of Medication Appropriateness intervention. Br J Clin Pharmacol 2022;88:2959–2968. 34913184 10.1111/bcp.15184

[61] Lakshmi R, James E, Kirthivasan R. Study on impact of clinical pharmacist's interventions in the optimal use of oral anticoagulants in stroke patients. Indian J Pharm Sci 2013;75:53. 23901161 10.4103/0250-474X.113550 PMC3719150

[62] Lee P-Y, Han SY, Miyahara RK. Adherence and outcomes of patients treated with dabigatran: pharmacist-managed anticoagulation clinic versus usual care. Am J Health Syst Pharm 2013;70:1154–1161. 23784163 10.2146/ajhp120634

[63] Liang J-B, Lao C-K, Tian L, Yang Y-Y, Wu H-M, Tong HH-Y et al. Impact of a pharmacist-led education and follow-up service on anticoagulation control and safety outcomes at a tertiary hospital in China: a randomised controlled trial. Int J Pharm Pract 2020;28:97–106. 31576625 10.1111/ijpp.12584

[64] Li X, Zuo C, Lu W, Zou Y, Xu Q, Li X et al. Evaluation of remote pharmacist-led outpatient service for geriatric patients on rivaroxaban for nonvalvular atrial fibrillation during the COVID-19 pandemic. Front Pharmacol 2020;11:1275. 32973511 10.3389/fphar.2020.01275 PMC7472570

[65] An T, Kose E, Kikkawa A, Hayashi H. Hospital pharmacist intervention improves the quality indicator of warfarin control: a retrospective cohort study. J Med Invest 2017;64:266–271. 28954994 10.2152/jmi.64.266

[66] Kose E, An T, Kikkawa A. Assessment of oral anticoagulation control at pharmacist-managed clinics: a retrospective cohort study. Pharmazie 2018;73:356–360. 29880089 10.1691/ph.2018.8322

[67] Kurimura T, Yamamoto K, Tanaka H, Toba T, Kimura T, Habu Y et al. Significance of pharmacist intervention to oral antithrombotic therapy in the pharmaceutical outpatient clinic of cardiovascular internal medicine: a retrospective cohort study. J Pharm Health Care Sci 2023;9:28. 37667376 10.1186/s40780-023-00296-9 PMC10478176

[68] Tarasiuk N, Parker M, Russo-Alvarez G, Cristiani C, Wai M. Hospital admission rates of patients enrolled in pharmacist vs nurse anticoagulation management services. J Am Coll Clin Pharm 2018;1:62–67.

[69] Noor A, Khan MA, Warsi A, Aseeri M, Ismail S. Evaluation of a pharmacist vs. haematologist-managed anticoagulation clinic: a retrospective cohort study. Saudi Pharm J 2021;29:1173–1180. 34703371 10.1016/j.jsps.2021.08.015 PMC8523325

[70] Manzoor BS, Bauman J, Shapiro NL, Stamos T, Galanter W, Nutescu EA. Outcomes of systematic anticoagulation management in pharmacist and nurse specialized clinics. J Am Coll Clin Pharm 2018;1:68–73.

[71] Derington CG, Goodrich GK, Xu S, Clark NP, Reynolds K, An J et al. Association of direct oral anticoagulation management strategies with clinical outcomes for adults with atrial fibrillation. JAMA Netw Open 2023;6:e2321971. 37410461 10.1001/jamanetworkopen.2023.21971 PMC10326649

[72] Falamić S, Lucijanić M, Ortner-Hadžiabdić M, Marušić S, Bačić-Vrca V. Pharmacists’ influence on adverse reactions to warfarin: a randomised controlled trial in elderly rural patients. Int J Clin Pharm 2019;41:1166–1173. 31493209 10.1007/s11096-019-00894-4

[73] DiRenzo BM, Beam DM, Kline JA, Deodhar KS, Weber ZA, Davis CM et al. Implementation and preliminary clinical outcomes of a pharmacist-managed venous thromboembolism clinic for patients treated with rivaroxaban post emergency department discharge. Acad Emerg Med 2018;25:634–640. 28921763 10.1111/acem.13311 PMC7089614

[74] Jones AE, King JB, Kim K, Witt DM. The role of clinical pharmacy anticoagulation services in direct oral anticoagulant monitoring. J Thromb Thrombolysis 2020;50:739–745. 32086703 10.1007/s11239-020-02064-0 PMC8744289

[75] Ritchie LA, Penson PE, Akpan A, Lip GYH, Lane DA. Integrated care for atrial fibrillation management: the role of the pharmacist. Am J Med 2022;135:1410–1426. 36002045 10.1016/j.amjmed.2022.07.014

[76] Fanikos J, Tawfik Y, Almheiri D, Sylvester K, Buckley LF, Dew C et al. Anticoagulation-associated adverse drug events in hospitalized patients across two time periods. Am J Med 2023;136:927–936.e3. 37247752 10.1016/j.amjmed.2023.05.013

[77] Higgins JP, Green S. Cochrane Handbook for Systematic Reviews of Interventions. 2008.

