# Supplementary material for: The effect of pharmacist-led interventions on the appropriateness and clinical outcomes of anticoagulant therapy: a systematic review and meta-analysis
Source: Eur Heart J Qual Care Clin Outcomes. 2024 Jul 13;10(6):488–506. doi: 10.1093/ehjqcco/qcae045 (PMC11873792; doi:10.1093/ehjqcco/qcae045)
Supplement: qcae045_Supplemental_Files [file qcae045_supplemental_files.zip › Supplementary Figures.docx]

**The Effect of Pharmacist-Led Interventions on the Appropriateness of Anticoagulation Therapy and Clinical Outcomes: A Systematic Review and Meta-Analysis**

**Supplementary Figures**

Panel A

Panel B

**Figure S1**: Subgroup analysis for heterogeneity in the appropriateness of anticoagulant therapy between pharmacist-led intervention and usual care groups (Panel A: based on type of medication prescribed, panel B: based on study region)

Panel A

Panel B

**Figure S2**: Sensitivity analysis for heterogeneity for anticoagulant therapy appropriateness between pharmacist-led intervention and usual care groups (panel A: one study excluded, panel B: two studies excluded)


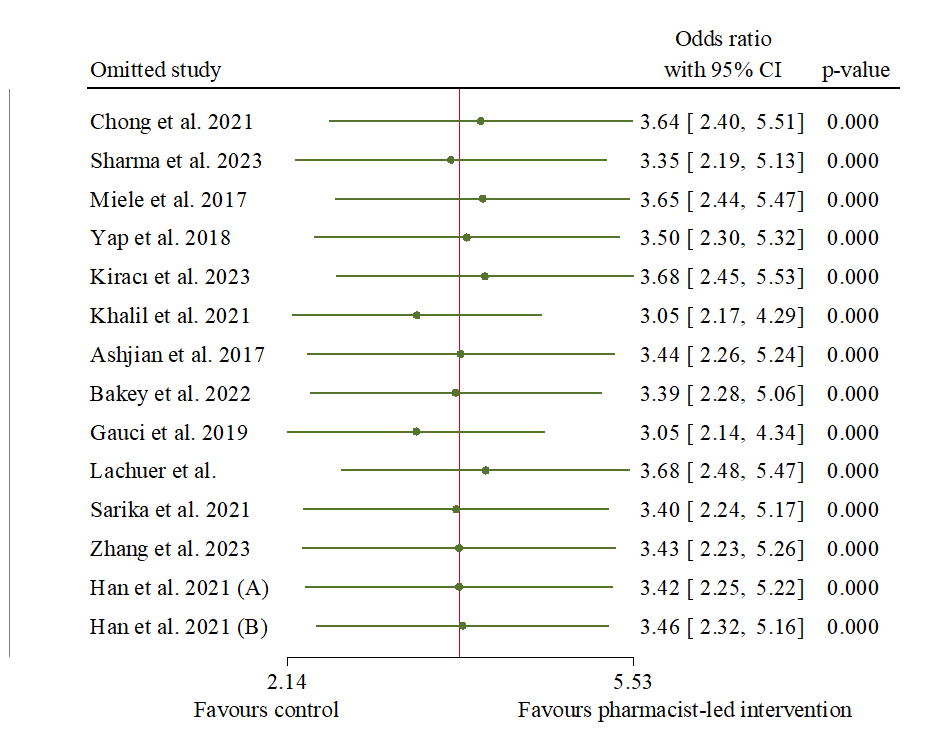


**Figure S3:** Summary estimates for anticoagulant appropriateness were calculated omitting one study at a time using a random effects model.

** Panel APanel B**

**Figure S4:** Subgroup analysis for heterogeneity of bleeding event between pharmacist-led intervention and usual care groups (Panel A: based on type of medication prescribed, panel B: based on study region).

**Figure S5**: Sensitivity analysis for heterogeneity and overall pooled estimate for bleeding events between pharmacist-led intervention and usual care groups (one study (Derington *et al*.) excluded).


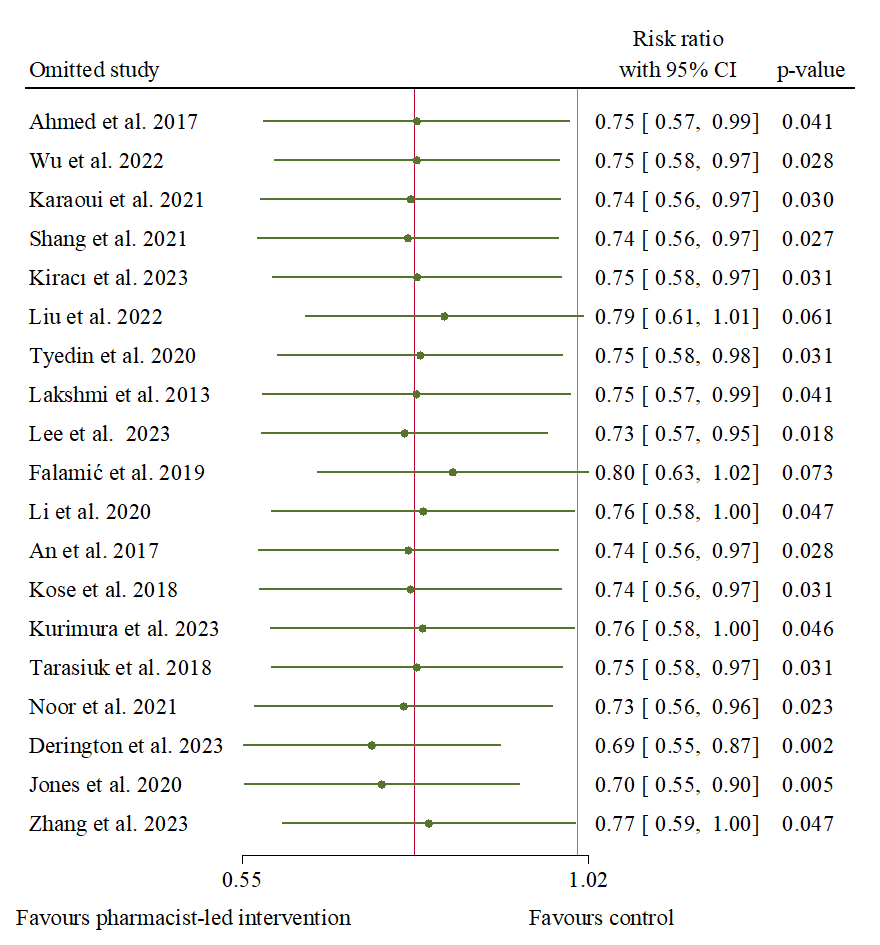


**Figure S6**: Summary estimates for bleeding were calculated omitting one study at a time using a random effects model.

Panel A

panel B

**Figure S7**: Major bleeding events (panel A) and minor bleeding events (panel B) between pharmacist-led intervention and usual care groups.

**Panel A**

**Panel B**

**Figure S8**: Subgroup analysis for heterogeneity of thromboembolic event between pharmacist-led intervention and usual care groups (Top panel: based on type of medication prescribed, bottom panel: based on study region).


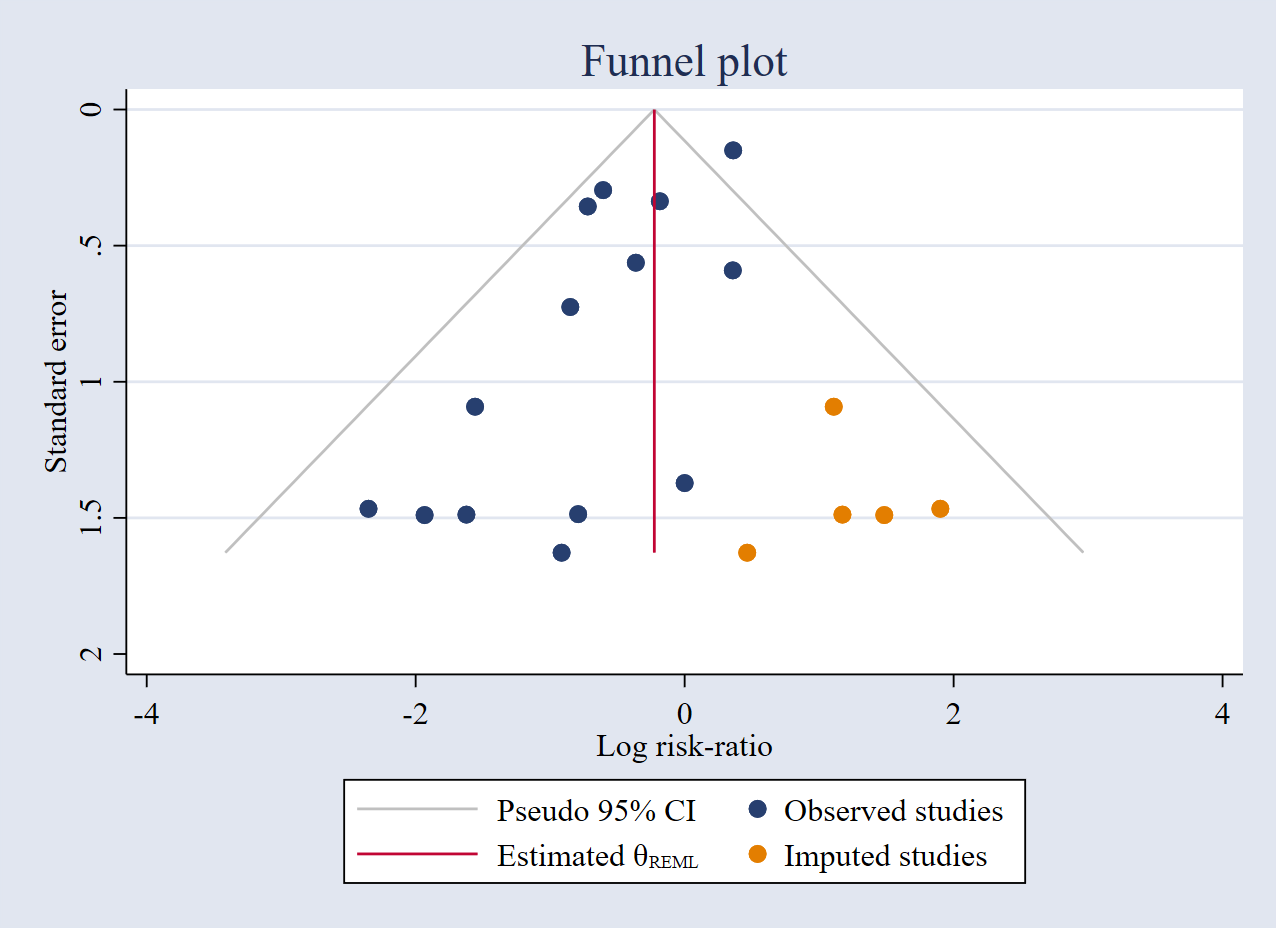


**Figure S9:** Trim-and-fill analysis for publication bias of thromboembolic event between pharmacist-led intervention and usual care groups.

**Figure S10:** Sensitivity analysis for heterogeneity and overall pooled estimate for thromboembolic events between pharmacist-led intervention and usual care groups (one study (Derington *et al*.) excluded).

**Figure S11**: Summary estimates for thromboembolic events were calculated omitting one study at a time using a random effects model.

Panel A

**Panel A**

**Panel B**

*Hospitalisation and/or emergency department visit

**Figure S12**: Subgroup analysis of hospitalisation between pharmacist-led intervention and usual care groups based on study regions (Panel A) and tpyes of medications prescribed (Panel B).

**Figure S13**: Summary estimates for hospitalisation were calculated omitting one study at a time using a random effects model.
