# Supplementary material for: The effect of pharmacist-led interventions on the appropriateness and clinical outcomes of anticoagulant therapy: a systematic review and meta-analysis
Source: Eur Heart J Qual Care Clin Outcomes. 2024 Jul 13;10(6):488–506. doi: 10.1093/ehjqcco/qcae045 (PMC11873792; doi:10.1093/ehjqcco/qcae045)
Supplement: qcae045_Supplemental_Files [file qcae045_supplemental_files.zip › Supplementary tables .docx]

**The Effect of Pharmacist-Led Interventions on the Appropriateness of Anticoagulation Therapy and Clinical Outcomes: A Systematic Review and Meta-Analysis**

**Supplementary tables**

**Table S1: study patient characteristics and inclusion and exclusion criteria**

| Study & year | Female, n (%) (C/I) | Age (mean, SD) (C/I) | Indications ACs | General initiative implemented | Inclusion criteria | Exclusion criteria |
| --- | --- | --- | --- | --- | --- | --- |
| Karaoui *et al. (1),* 2021 | 57 (57) | 73.2 (14.7) | AF, VTE treatment, Aortic VR, Mitral VR | Pharmacist AC therapy education on bleeding and readmission rates | Inpatients aged ≥18 years, admitted to the hospital, and discharged on an oral AC for a therapeutic indication | Patients with severe cognitive impairment, unstable psychiatric illness, inability to communicate in Arabic/English, inability to be followed-up, and patients discharged on an AC for VTE prophylaxis, or otherwise being too ill to participate. |
|  | 54 (54) | 74.7 (12.1) |  |  |  |  |
| Liu et al. (10), 2022 | 35 (54.7) | 61.9 (11.5) | DVT | Post-discharge pharmacist-led follow-up in patients with DVT | Patients diagnosed with DVT, who were discharged after treatment in the acute phase, treated with rivaroxaban or warfarin after discharge and participation in the study voluntary | Patients with psychiatric disorder, dysopia or dysaudia. |
|  | 28 (45.9) | 63.8 (12.1) |  |  |  |  |
| Lakshmi et al. (15), 2013 | 16 (40) | 56 | AF, VTE, and VR | CP interventions in optimising OACs use in stroke Patients | Patients prescribed OACs; age ≥18 years; willing to participate in the study; can read and speak English or the local language Malayalam | Patients on parenteral ACs; with severe renal insufficiency who are on dialysis; with active liver disease; having visual or hearing impairment |
|  | 16 (40)) | 56 |  |  |  |  |
| Liang et al. (17), 2020 | 31 (41.3) | 62.5 (14.5) | AF, DVT, PE | Pharmacist-led anticoagulation service on AC control | Patients with aged ≥ 18 years and newly initiated on warfarin during hospitalisation, with intended treatment duration of at least 3 months | Participants who died or were switched to other anticoagulants before discharge and who had no INR measurement following discharge |
|  | 36 (46.8) | 60.1 (16.3) |  |  |  |  |
| Falamić et al. (18), 2019 | 32 (48%) | 75 (71–81)) | AF, Thrombophlebitis | Community pharmacists’ intervention on the risk of ADRs in elderly patients with warfarin | Age ≥65 years, rural residence, and duration of warfarin therapy of at least 3 months before the study with an expected duration of at least a further 6 months | Patients with prescribed interacting drug with X degree of clinical significance based on the Lexi®-interact database. |
|  | 32 (48%) | 72 (68–79) |  |  |  |  |
| Chong et al. (1), 2021 | 182 (46) | 71 (17) | VTE prophylaxis | VTE stewardship program across four hospitals | Adult medical and surgical patients with length of stay >24 hrs. | Patients in the emergency department, day surgery unit, intensive care unit, palliative care, maternity, and mental health wards. |
|  | 194 (47) | 72 (17) |  |  |  |  |
| Ahmed et al. (2), 2017 | 99 (73.3) | 41.8 (13) | VR | CP intervention in patients with warfarin | Adult patients on warfarin therapy for at least the previous year, on regular follow up at the hospital, and has a complete follow up data for the past 12 months (12 INR readings) | Pregnant women, patients who cannot communicate in Arabic language or those who have hearing or cognitive problems |
|  | 99 (73.3 | 41.8 (13) |  |  |  |  |
| Wu et al. (3), 2022 | 13 (33.3) | 47.4 ± 13.6 | VR | Pharmacist management of warfarin initiation in post-mechanical VR patients | Patients ≥20 years of age and admitted for mechanical VR with newly started warfarin therapy during the study period | Patients received warfarin within 1 year before surgery and had embolic or haemorrhagic stroke caused by infective endocarditis before surgery |
|  | 6 (18.2) | 47.6 ± 13.4 |  |  |  |  |
| Sharma et al. (4), 2023 | 213 (45.3) | 67.5 | AF | Pharmacist-led interventions for optimising OAC therapies in AF patients | Patients aged ≥18 years with a confirmed AF diagnosis and on the Quality and Outcomes Framework AF register | Patients with confirmed diagnosis of valvular AF |
|  | NR | NR |  |  |  |  |
| Miele et al. (5), 2017 | 25 (50) | 77.0 (1.5) | VTE  AF | Pharmacist driven DOAC protocol | Patients ≥18 years, and receiving a DOAC for a minimum of 2 consecutive days during the study period for an indication of nonvalvular AF and/or VTE treatment/prophylaxis | Patients with missing data that would interfere with the ability to assess for DOAC appropriateness |
|  | 48 (56) | 69.6 (1.6) |  |  |  |  |
| Yap et al. (7), 2018 | NR | NR | DVT prophylaxis | Pharmacist driven DVT Risk Alert Tool | Patients admitted to medical wards during the study period | Patient who received therapeutic or prophylactic ACs prior to the current admission, hospitalised <24 hours, contraindicated to DVT prophylaxis treatment, and received therapeutic doses of AC for therapeutic purposes |
|  | 96 (44) | 72.1 (12.8) |  |  |  |  |
| Shang et al. (8), 2021 | 158 (65.8 | 72.8 (11.5) | VTE prophylaxis | CP services on the rationality of AC therapy and the incidence of thrombosis. | Patients with THA/TKA procedure during the study period, the preoperative Doppler ultrasonography of both lower limbs confirmed negative, the electronic medical records were complete | Patients with planned revision arthroplasty, intra-articular debridement, postoperative infection, antibiotic bone cement, positive preoperative Doppler ultrasonography, long-term antithrombotic therapy, and missing or incomplete electronic medical records. |
|  | 240 (71.2) | 70.1 (11.8) |  |  |  |  |
| Kiracı et al. (9), 2023 | 182 (53.5) | 51.1 (14.7) | VTE prophylaxis | CP services and education on VTE risk and thromboprophylaxis appropriateness | Patients aged ≥18 years who had a hospitalisation plan of at least 24hrs, hospitalised with an indication for surgery, conscious preoperatively or accompanied by a first-degree relative | Patients who were on any ACs drug, had bleeding tendency, were not followed up in general surgery wards, had a hospitalisation period of <24 hrs and did not give consent to participate |
|  | 118 (61.8) | 54.8 (15.1) |  |  |  |  |
| Khalil et al. (11), 2021 | 30 (48) | M= 75.30  F= 74.60 | AF | Pharmacist-led shared decision making on AC therapy appropriateness in AF patients | All patients (≥ 18 years of age) with newly diagnosed and admitted AF, expected length of stay > 24hrs and consented to participate in a satisfaction survey | Patients admitted with pre-existing AF; a new diagnosis of AF but not considered for AC; or if they were not able to communicate due to language barrier, severe illness, or cognitive impairment |
|  | 29 (45) | M= 72.78  F= 75.03 |  |  |  |  |
| Tyedin et al. (12), 2020 | 56 (43.1) | 74.4 (13.4) | AF, DVT, PE, VR | Pharmacist-assisted electronic warfarin charting and monitoring | Patients aged ≥18 years, admitted to an inpatient study ward for at least 24 h and prescribed at least one warfarin dose during their admission | NR |
|  | 40 (37) | 70.0 (16.1) |  |  |  |  |
| Hyland et al. (13), 2020 | NR | NR | THA/TKA | An orthopaedic CP service on institutional total joint arthroplasty | All adult patients admitted to the study institution for a TKA or THA procedure during the study period | NR |
|  | NR | NR |  |  |  |  |
| Quintens et al. (14), 2021 | 280 (60.1) | 71.1 (17.3) | AF, VTE prophylaxis | Check of Medication Appropriateness on AC prescribing | Non-critically ill inpatients admitted to wards | NR |
|  | 254 (52.4) | 75.2 (14.8) |  |  |  |  |
| Lee et al. (16), 2013 | 1 (2) | 72 (67–81) | AF or atrial flutter | Pharmacist monitoring during early dabigatran therapy for AF -patients | Patients with newly initiated dabigatran on a twice-daily dosing regimen for the management of nonvalvular AF or flutter during the study period. | Patients with dabigatran initiation were too recent (i.e., they had not yet completed three months of follow-up) or therapy was discontinued within three months of initiation. Patients who received any ACC services within the three-month study period. |
|  | 0 (0) | 78 (72–83) |  |  |  |  |
| Gauci et al. (30), 2019 | 96 (64) | 81.7 (7.6) | AF | CP intervention or implementation of a developed MAT-AF tool | Patients with a diagnosis of AF and age ≥60 years | Patients transfer to acute care and death |
|  | 106 (70.7) | 82.7 (6.4) |  |  |  |  |
| Sarika et al. (32), 2021 | 8 (17.8) | 60.49 (11.7) | IHD | CP directed AC monitoring services in cardiology department | Patients with age >18 years, and those who had received at least one prescription order for heparin | Patients who had voluntarily discharged themselves, lactating mothers, those with missing data, patients of non-Indian origin and pregnant patients |
|  | 11(24.4) | 62.6 (11.8) |  |  |  |  |
| Lachuer et al. (35), 2021 | 34 (58.6) | 86.3 | Any indication of OAC | Geriatricians' training on the improvement of their prescribing practices | Prescriptions of all patients treated with VKA or DOAC and hospitalised | NR |
|  | 34 (58.6) | 86.3 |  |  |  |  |
| Li et al. (19), 2020 | 80 (39.6) | 75.2(7.1) | Non-valvular AF | Pharmacists’ rivaroxaban management for geriatric patients with nonvalvular AF | Age ≥65 years, high risk for stroke, TIA, and systemic embolism in the CHA2DS2-VASc score (≥1) and received rivaroxaban for at least one month. | History of bleeding and haemorrhagic diseases, prescribed with another anticoagulant, severe renal dysfunction and unable to communicate via WeChat or phone. |
|  | 69 (38.5) | 76.3(7.8) |  |  |  |  |
| DiRenzo *et al*. (2), 2018 | 11 (65) | 54 (12) | VTE prophylaxis | Pharmacist-managed clinic for VTE treatment | Low-risk VTE | NR |
|  | 11 (65) | 50 (12) |  |  |  |  |
| An *et al*. (3), 2017 | 9 (28.1) | 72 (66-77) | NVAF | TTR control in nonvalvular AF patients receiving warfarin | Patients admitted to the cardiovascular medicine department for arrhythmia ablation, who started warfarin therapy, had a warfarin duration of over a year, and had heart failure. | Patients with missing data and those who had NVAF without heart failure |
|  | 13 (52) | 70 (64-77) |  |  |  |  |
| Ashjian *et al. (4),* 2017 | 58 (45) | 66 (56–75) | AF  VTE | Pharmacist-led DOAC service | Patients age ≥18 years or older who participated in an encounter with a pharmacist as part of the AMS- DOAC service during the study period | Patients receiving haemodialysis and if their DOAC prescription was never filled based on their pharmacy records despite the notation of an active pre­scription in the EMR. |
|  | 61 (47) | 67 (58–75) |  |  |  |  |
| Kose *et al. (5),* 2018 | 19 (18) | 72.3 (1.8) | NVAF | CP interventions on TTR control for chronic kidney disease patients with NVAF on warfarin | Patients admitted to the cardiovascular medicine department for arrhythmia ablation who started warfarin therapy, had a warfarin duration of over a year, and had CKD and PT-INR measured at least once every 1–2. | Patients with missing data and those who had NVAF without CKD |
|  | 7 (44) | 71.8 (2.2) |  |  |  |  |
| Bakey *et al*. (6), 2022 | 18 (40.9) | 50.5 (43-62.5) | VTE | Pharmacist involvement in low risk VTE patients with DOAC | NR | Non-acute VTE, inaccurate diagnosis, required hospital admission for any non-VTE reason, or the VTE was diagnosed while inpatient. |
|  | 6 (42.9) | 61.0 (44-69) |  |  |  |  |
| Kurimura *et al. (7),* 2023 | 76 (28.8) | 72.6 (11.8) | AF  IHD  DVT | Pharmacist intervention for outpatients taking antithrombotic drugs | Patients who visited the outpatient clinic of cardiovascular internal medicine during the study period and  taking antithrombotic drugs. | NR |
|  | 38 (28.8) | 73.2 (12.2) |  |  |  |  |
| Tarasiuk *et al*. (8), 2018 | 135 (56.3) | 63.5 (15.3) | AF and VTE | Pharmacists-managed AMS | Patients age ≥18 years and had an anticoagulation consultation during the enrolment period | Patients had a lapse of 10 weeks without a point-of-care INR draw or had a duration of warfarin management less than 2 weeks. |
|  | 118 (51.8) | 65.2 (16.4) |  |  |  |  |
| Noor *et al. (9),* 2021 | 80 (64.5) | 57.9 (19.2) | DVT, PE, AF,  Mechanical VR Antiphospholipid syndrome | Pharmacist-managed anticoagulation clinic in the Ambulatory Care Centre | Patients (≥18 years), with regular follow-up visits at the anticoagulation clinic for at least six months and receiving warfarin for an extended duration for any of the following indications: DVT, PE, AF, mechanical  VR, and antiphospholipid syndrome. | Pregnant women and patients for whom the target therapeutic INR was increased due to the development of a new clinical condition; INR results of patients within the first 30 days of the initiation of warfarin or post-discharge in cases of hospitalisation and INR results during a temporary planned interruption |
|  | 58 (55.7) | 64.7 (13.8) |  |  |  |  |
| Manzoor *et al*. (10), 2018 | 52 (52) | 64.2 (13.2) | PVD, VTE, stroke, AF, mechanical VR | Pharmacist-managed clinics | Patients >18 years of age and being treated with warfarin for a minimum of 1 month | NR |
|  | 65 (65) | 58.7 (15.5) |  |  |  |  |
| Derington *et al. (11),* 2023 | 2783 (45) | 72.5 | AF or flutter | OAC-care models for preventing adverse AC-related outcomes among AF patients | NR | Patients with a history of OAC use in the past 6 months, dispensed more than 1 OAC on the index date, died prior to the index date, were <18 years of age on the index date, had less than 365 days of KP health plan membership prior to the index date, and did not have a diagnosis of AF within 1 year before or 7 days after the index date. |
|  | 2258 (45.7) | 73.7 |  |  |  |  |
| Jones *et al. (12),* 2020 | 141 (38.1) | 67.1 (12.0) | AF | DOAC management by AMS | NR | Patients with insufficient information to determine study outcomes, not receiving DOAC therapy, and cases where DOAC therapy was managed outside the healthcare system. |
|  | 34 (37.8) | 68.9 (11.0) |  |  |  |  |
| Zhang *et al. (13),* 2023 | 63 (38.9) | 68 (56-77) | VTE  Prophylaxis | CPs on ICU physicians' compliance with VTE guidelines | Length of hospital stays ≥ 72 hours.  Age ≥ 16 years. | Patients who had been diagnosed with PE or DVT on admission.  Patients were receiving anticoagulation therapy. |
|  | 77 (43.8) | 66 (60-77) |  |  |  |  |
| Han *et al*. *(14),* 2021 | 51 (37.2) | 69 (63–76) | AF, VTE, CAD, PAD, and mechanical heart valve | Implementation EMR-based alert | Patients ≥18 years old and taking OAC or an antiplatelet agent were also ordered for outpatient elective GI endoscopy. | Patients without anticoagulant or antiplatelet medication, inpatient, or urgent endoscopy needs, or only taking aspirin, were excluded from the procedure. |
|  | 233 (42.3) | 68 (61–75) |  |  |  |  |

AC = anticoagulant; AMS = anticoagulation management service; INR: international normalised ratio; PT-INR: prothrombin time-INR; CP: clinical pharmacist; DOAC: direct acting oral anticoagulant; DTI: direct thrombin inhibitor; OAC: oral anticoagulant; VKA: vitamin K antagonist; CAD: coronary artery disease; CKD: chronic kidney disease, DVT: deep venous thrombosis; IHD: ischemic heart disease; PAD: peripheral arterial disease PE: pulmonary embolism; PVD: peripheral vascular disease; TE: thromboembolic; VTE: venous thromboembolism; VR: valve replacement; THA/TKA: total hip arthroplasty/total knee arthroplasty.

**Table S2: Assessment of the included Randomised Controlled Trials (RCTs) using Joanna Briggs Institute (JBI) critical appraisal tool.**

| Studies | JBI Critical Appraisal Checklists for RCTs (Response: Yes, No, Unclear or Not applicable) | | | | | | | | | | | | | Total score | Study quality |
| --- | --- | --- | --- | --- | --- | --- | --- | --- | --- | --- | --- | --- | --- | --- | --- |
|  | Q1 | Q2 | Q3 | Q4 | Q5 | Q6 | Q7 | Q8 | Q9 | Q10 | Q11 | Q12 | Q13 |  |  |
| Karaoui *et al*. (1) | Y | Y | Y | N | N | Y | N | Y | U | Y | Y | Y | Y | 69.2% | Moderate |
| Liu *et al*. (15) | Y | Y | Y | U | U | Y | N | Y | U | Y | Y | Y | U | 61.5% | Moderate |
| Lakshmi *et al*. (16) | N | U | Y | N | N | Y | U | Y | Y | Y | Y | Y | Y | 61.5% | Moderate |
| Liang *et al*. (17) | Y | Y | Y | Y | U | Y | Y | Y | Y | Y | Y | Y | Y | 92.3% | High |
| Falamić *et al*. (18) | Y | U | Y | U | U | Y | U | Y | Y | Y | Y | Y | Y | 69.2% | Moderate |

Scores can range from 0 to 13, each question is given a single score for yes and 0 for no, unclear, and not applicable (NA); High quality: % of score >70, Moderate quality: % of score 50-70, Low quality: % of score <50

Q1: Was true randomization used for assignment of participants to treatment groups? Q2: Was allocation to treatment groups concealed? Q3: Were treatment groups similar at the baseline? Q4: Were participants blind to treatment assignment? Q5: Were those delivering the treatment blind to treatment assignment? Q6: Were treatment groups treated identically other than the intervention of interest? Q7: Were outcome assessors blind to treatment assignment? Q8: Were outcomes measured in the same way for treatment groups? Q9: Were outcomes measured in a reliable way? Q10: Was follow up complete and if not, were differences between groups in terms of their follow up adequately described and analysed? Q11: Were participants analysed in the groups to which they were randomized? Q12: Was appropriate statistical analysis used? Q13: Was the trial design appropriate and any deviations from the standard RCT design (individual randomization, parallel groups) accounted for in the conduct and analysis of the trial?

**Table S3: Assessment of quasi-experimental studies (i.e., before-after or non-randomized study designs) using Joanna Briggs Institute (JBI) critical appraisal tool.**

| Studies | JBI Critical Appraisal Checklists for Quasi-Experimental Studies (Response: Yes, No, Unclear or Not applicable) | | | | | | | | | Total score | Study quality |
| --- | --- | --- | --- | --- | --- | --- | --- | --- | --- | --- | --- |
|  | Q1 | Q2 | Q3 | Q4 | Q5 | Q6 | Q7 | Q8 | Q9 |  |  |
| Chong *et al. (19)* | Y | Y | Y | N | Y | Y | Y | Y | Y | 88.9% | High |
| Ahmed *et al*. (20) | Y | Y | Y | N | Y | Y | Y | N | Y | 77.8% | High |
| Wu *et al*. (21) | Y | Y | Y | N | N | Y | Y | N | Y | 66.7% | Moderate |
| Sharma *et al*. (22) | Y | Y | Y | N | N | Y | Y | Y | Y | 77.8% | High |
| Miele *et al*. (23) | Y | Y | Y | N | N | Y | Y | Y | Y | 77.8% | High |
| Yap *et al*. (24) | Y | Y | Y | N | N | Y | Y | Y | Y | 77.8% | High |
| Shang *et al*. (25) | Y | N | Y | N | Y | Y | Y | Y | Y | 77.8% | High |
| Kiracı *et al*. (26) | Y | N | Y | N | Y | Y | Y | Y | Y | 77.8% | High |
| Khalil *et al*. (27) | Y | Y | Y | N | Y | N | Y | Y | Y | 77.8% | High |
| Tyedin *et al*. (28) | Y | N | N | N | Y | Y | Y | Y | Y | 66.7% | Moderate |
| Hyland *et al*. (29) | Y | N | Y | N | Y | Y | Y | Y | Y | 77.8% | High |
| Quintens *et al*. (30) | Y | N | Y | N | Y | Y | Y | Y | Y | 77.8% | High |
| Lee *et al*. (31) | Y | Y | Y | N | Y | N | Y | Y | Y | 77.8% | High |
| Gauci *et al*. (32) | Y | Y | Y | N | N | Y | Y | Y | Y | 77.8% | High |
| Sarika *et al*. (33) | Y | Y | Y | N | N | Y | Y | Y | Y | 77.8% | High |
| Lachuer *et al*. (34) | Y | Y | Y | Y | Y | Y | Y | Y | Y | 100% | High |

Scores can range from 0 to 9, each question is given a single score for yes and 0 for no, unclear, and not applicable (NA); High quality: % of score >70, Moderate quality: % of score 50-70, Low quality: % of score <50

Q1: Is it clear in the study what is the ‘cause’ and what is the ‘effect’ (i.e. there is no confusion about which variable comes first)? Q2: Were the participants included in any comparisons similar? Q3: Were the participants included in any comparisons receiving similar treatment/care, other than the exposure or intervention of interest? Q4: Was there a control group? Q5: Were there multiple measurements of the outcome both pre and post the intervention/exposure? Q6: Was follow up complete and if not, were differences between groups in terms of their follow up adequately described and analysed? Q7: Were the outcomes of participants included in any comparisons measured in the same way? Q8: Were outcomes measured in a reliable way? Q9: Was appropriate statistical analysis used?

**Table S4: Assessment of cohort studies using Joanna Briggs Institute (JBI) critical appraisal tool**

| Studies | JBI Critical Appraisal Checklists for RCTs (Response: Yes, No, Unclear or Not applicable) | | | | | | | | | | | Total score | Study quality |
| --- | --- | --- | --- | --- | --- | --- | --- | --- | --- | --- | --- | --- | --- |
|  | Q1 | Q2 | Q3 | Q4 | Q5 | Q6 | Q7 | Q8 | Q9 | Q10 | Q11 |  |  |
| Li *et al*. (35) | Y | Y | Y | N | N | Y | N | Y | Y | Y | Y | 72.7% | High |
| DiRenzo *et al*. (2) | Y | Y | Y | N | N | Y | Y | Y | Y | Y | Y | 81.8% | High |
| An *et al*. (3) | Y | Y | N | N | N | Y | Y | Y | Y | Y | Y | 72.7% | High |
| Ashjian *et al*. (4) | N | Y | Y | N | N | Y | Y | Y | Y | Y | Y | 72.7% | High |
| Kose *et al*. (5) | Y | Y | N | N | N | Y | Y | Y | Y | Y | Y | 72.7% | High |
| Bakey *et al*. (6) | Y | Y | Y | N | N | Y | Y | Y | Y | N | Y | 72.7% | High |
| Kurimura *et al*. (7) | Y | Y | Y | Y | Y | Y | Y | Y | Y | N | Y | 90.9% | High |
| Tarasiuk *et al*. (8) | N | Y | Y | Y | Y | Y | Y | Y | Y | N | Y | 81.8% | High |
| Noor *et al*. (9) | N | Y | Y | Y | Y | Y | Y | Y | Y | N | Y | 81.8% | High |
| Manzoor *et al*. (10) | N | Y | Y | Y | Y | Y | Y | Y | Y | N | Y | 81.8% | High |
| Derington *et al*. (11) | N | U | Y | Y | U | Y | Y | Y | Y | NA | Y | 63.6% | Moderate |
| Jones *et al*. (12) | N | Y | Y | Y | N | Y | Y | Y | Y | Y | Y | 81.8% | High |
| Zhang *et al*. (13) | Y | Y | Y | Y | Y | Y | Y | Y | Y | NA | Y | 90.9% | High |
| Han *et al*. (14) | N | Y | Y | Y | Y | Y | Y | Y | Y | NA | Y | 81.8% | High |

Scores can range from 0 to 11, each question is given a single score for yes and 0 for no, unclear, and not applicable (NA); High quality: % of score >70, Moderate quality: % of score 50-70, Low quality: % of score <50

Q1: Were the two groups similar and recruited from the same population? Q2: Were the exposures measured similarly to assign people to both exposed and unexposed groups? Q3: Was the exposure measured in a valid and reliable way? Q4: Were confounding factors identified? Q5: Were strategies to deal with confounding factors stated? Q6: Were the groups/participants free of the outcome at the start of the study (or at the moment of exposure)? Q7: Were the outcomes measured in a valid and reliable way? Q8: Was the follow up time reported and sufficient to be long enough for outcomes to occur? Q9: Was follow up complete, and if not, were the reasons to loss to follow up described and explored? Q10: Were strategies to address incomplete follow up utilized? Q11: Was appropriate statistical analysis used?

**References**

1. Karaoui LR, Ramia E, Mansour H, Haddad N, Chamoun N. Impact of pharmacist-conducted anticoagulation patient education and telephone follow-up on transitions of care: a randomized controlled trial. *BMC Health Services Research*. 2021;21:1-12.

2. DiRenzo BM, Beam DM, Kline JA, Deodhar KS, Weber ZA, Davis CM, et al. Implementation and Preliminary Clinical Outcomes of a Pharmacist‐managed Venous Thromboembolism Clinic for Patients Treated With Rivaroxaban Post Emergency Department Discharge. *Academic Emergency Medicine*. 2018;25(6):634-40.

3. An T, Kose E, Kikkawa A, Hayashi H. Hospital pharmacist intervention improves the quality indicator of warfarin control: A retrospective cohort study. *The Journal of Medical Investigation*. 2017;64(3.4):266-71.

4. Ashjian E, Kurtz B, Renner E, Yeshe R, Barnes GD. Evaluation of a pharmacist-led outpatient direct oral anticoagulant service. *American Journal of Health-System Pharmacy*. 2017;74(7):483-9.

5. Kose E, An T, Kikkawa A. Assessment of oral anticoagulation control at pharmacist-managed clinics: A retrospective cohort study. *Die Pharmazie-An International Journal of Pharmaceutical Sciences*. 2018;73(6):356-60.

6. Bakey KH, Nguyen C-TN. Impact of a pharmacist intervention in the emergency department on the appropriateness of direct oral anticoagulants prescribed in venous thromboembolism patients. *Journal of Pharmacy Practice*. 2022;35(4):599-605.

7. Kurimura T, Yamamoto K, Tanaka H, Toba T, Kimura T, Habu Y, et al. Significance of pharmacist intervention to oral antithrombotic therapy in the pharmaceutical outpatient clinic of cardiovascular internal medicine: a retrospective cohort study. *Journal of Pharmaceutical Health Care and Sciences*. 2023;9(1):28.

8. Tarasiuk N, Parker M, Russo‐Alvarez G, Cristiani C, Wai M. Hospital admission rates of patients enrolled in pharmacist vs nurse anticoagulation management services. *Journal of the American College of Clinical Pharmacy*. 2018;1(2):62-7.

9. Noor A, Khan MA, Warsi A, Aseeri M, Ismail S. Evaluation of a pharmacist vs. Haematologist-managed anticoagulation clinic: A retrospective cohort study. *Saudi Pharmaceutical Journal*. 2021;29(10):1173-80.

10. Manzoor BS, Bauman J, Shapiro NL, Stamos T, Galanter W, Nutescu EA. Outcomes of systematic anticoagulation management in pharmacist and nurse specialized clinics. *Journal of the American College of Clinical Pharmacy*. 2018;1(2):68-73.

11. Derington CG, Goodrich GK, Xu S, Clark NP, Reynolds K, An J, et al. Association of Direct Oral Anticoagulation Management Strategies With Clinical Outcomes for Adults With Atrial Fibrillation. *JAMA Network Open*. 2023;6(7):e2321971-e.

12. Jones AE, King JB, Kim K, Witt DM. The role of clinical pharmacy anticoagulation services in direct oral anticoagulant monitoring. *Journal of Thrombosis and Thrombolysis*. 2020;50(3):739-45.

13. Zhang L, Wang Y, Zhang K, Li P, Qiao Y, Wang H, et al. Impact of clinical pharmacist services on physicians' guideline compliance and prognosis of patients for venous thromboembolism prophylaxis in ICU. *International Journal of Clinical Pharmacology and Therapeutics*. 2022.

14. Han H, Chung G, Sippola E, Chen W, Morgan S, Renner E, et al. Improving preprocedure antithrombotic management: Implementation and sustainment of a best practice alert and pharmacist referral process. *Research and Practice in Thrombosis and Haemostasis*. 2021;5(5):e12558.

15. Liu X, Xiao Q, Li Y, Hu G, Kun W, Xu W. Effect of post-discharge pharmacist-led follow-up on drug treatment in patients with deep venous thrombosis in primary hospitals in China. *Pakistan Journal of Pharmaceutical Sciences*. 2022;35(3).

16. Lakshmi R, James E, Kirthivasan R. Study on impact of clinical pharmacist’s interventions in the optimal use of oral anticoagulants in stroke patients. *Indian Journal of Pharmaceutical Sciences*. 2013;75(1):53.

17. Liang J-B, Lao C-K, Tian L, Yang Y-Y, Wu H-M, Tong HH-Y, et al. Impact of a pharmacist-led education and follow-up service on anticoagulation control and safety outcomes at a tertiary hospital in China: a randomised controlled trial. *International Journal of Pharmacy Practic*e. 2020;28(1):97-106.

18. Falamić S, Lucijanić M, Ortner-Hadžiabdić M, Marušić S, Bačić-Vrca V. Pharmacists’ influence on adverse reactions to warfarin: a randomised controlled trial in elderly rural patients. *International Journal of Clinical Pharmacy*. 2019;41:1166-73.

19. Chong J, Curtain C, Gad F, Passam F, Soo G, Levy R, et al. Development and implementation of venous thromboembolism stewardship across a hospital network. *International Journal of Medical Informatics*. 2021;155:104575.

20. Ahmed NO, Osman B, Abdelhai YM, El-Hadiyah TMH. Impact of clinical pharmacist intervention in anticoagulation clinic in Sudan. *International Journal of Clinical Pharmacy*. 2017;39:769-73.

21. Wu C-W, Wu C-C, Chen C-H, Lin S-Y, Hsu R-B, Huang C-F. The impact of pharmacist-managed service on warfarin therapy in patients after mechanical valve replacement. *International Journal of Clinical Practice*. 2022;2022.

22. Sharma R, Hasan SS, Gilkar IA, Hussain WF, Conway BR, Ghori MU. Pharmacist-Led interventions in optimising the use of oral anticoagulants in atrial fibrillation patients in the general practice in England: A retrospective observational study. *BJGP open*. 2023.

23. Miele C, Taylor M, Shah A. Assessment of direct oral anticoagulant prescribing and monitoring pre-and post-implementation of a pharmacy protocol at a community teaching hospital. *Hospital Pharmacy*. 2017;52(3):207-13.

24. Yap DFS, Ng ZY, Wong CY, Saifuzzaman MM, Yang L. Appropriateness of deep vein thrombosis (DVT) prophylaxis use among medical inpatients: a DVT risk alert tool (DRAT) study. *Medical Journal of Malaysia*. 2019;74(1):45.

25. Shang J, Ning W, Gong J, Su D, Jia X, Wang Y. Impact of clinical pharmacist services on anticoagulation management of total joint arthroplasty: A retrospective observational study. *Journal of Clinical Pharmacy and Therapeutics*. 2021;46(5):1301-7.

26. Kiracı ZK, Yalçın N, Cennet Ö, Demirkan K, Yorgancı K. Education and clinical pharmacist-led management strategies for the risk and prophylaxis of venous thromboembolism in general surgery. *Thrombosis Journal*. 2023;21(1):86.

27. Khalil V, Blackley S, Subramaniam A. Evaluation of a pharmacist-led shared decision-making in atrial fibrillation and patients’ satisfaction—a before and after pilot study. *Irish Journal of Medical Science (1971-)*. 2021;190:819-24.

28. Tyedin AE, Taylor SE, Than J, Al‐Alawi R, O’Halloran E, Chau AH. Impact of proactive pharmacist‐assisted warfarin management using an electronic medication management system in Australian hospitalised patients. *Journal of Pharmacy Practice and Research*. 2020;50(2):144-51.

29. Hyland SJ, Kramer BJ, Fada RA, Lucki MM. Clinical pharmacist service associated with improved outcomes and cost savings in total joint arthroplasty. *The Journal of Arthroplasty*. 2020;35(9):2307-17. e1.

30. Quintens C, Verhamme P, Vanassche T, Vandenbriele C, Van den Bosch B, Peetermans WE, et al. Improving appropriate use of anticoagulants in hospitalised patients: A pharmacist‐led Check of Medication Appropriateness intervention. *British Journal of Clinical Pharmacology*. 2022;88(6):2959-68.

31. Lee P-Y, Han SY, Miyahara RK. Adherence and outcomes of patients treated with dabigatran: pharmacist-managed anticoagulation clinic versus usual care. *American Journal of Health-System Pharmacy*. 2013;70(13):1154-61.

32. Gauci M, Wirth F, Azzopardi LM, Serracino-Inglott A. Clinical pharmacist implementation of a medication assessment tool for long-term management of atrial fibrillation in older persons. *Pharmacy Practice (Granada)*. 2019;17(1).

33. Sarika A, Reghu A, Karattuthodi MS, Sreelatha ARP. Clinical pharmacist directed anticoagulation monitoring services: A prospective interventional study. *İstanbul Journal of Pharmacy*. 2021;51(3):291-8.

34. Lachuer C, Benzengli H, Do B, Rwabihama J-P, Leglise P, editors. Oral anticoagulants: Interventional pharmaceutical study with reminder of good practices, and iatrogenic impact. *Annales Pharmaceutiques Françaises*; 2021: Elsevier.

35. Li X, Zuo C, Lu W, Zou Y, Xu Q, Li X, et al. Evaluation of remote pharmacist-led outpatient service for geriatric patients on rivaroxaban for nonvalvular atrial fibrillation during the COVID-19 pandemic. *Frontiers in Pharmacology*. 2020;11:1275.
